# Expressions of Olfactory Proteins in Locust Olfactory Organs and a Palp Odorant Receptor Involved in Plant Aldehydes Detection

**DOI:** 10.3389/fphys.2018.00663

**Published:** 2018-06-04

**Authors:** Hongwei Li, Peng Wang, Liwei Zhang, Xiao Xu, Zewen Cao, Long Zhang

**Affiliations:** Department of Entomology, China Agricultural University, Beijing, China

**Keywords:** olfactory organs, electrophysiological response, odorant binding protein, odorant receptors, *Locusta migratoria*

## Abstract

The main chemosensory organs of locusts consisted of the antennae and the mouthparts (maxillary and labial palps), which are suggested to perform different functions. However, very few are known about the differences of these two organs at molecular level. To understand the differences of locust antennae and palps in olfaction, the electrophysiological response and olfactory gene expression of these two organs were conducted. Our electrophysiological experiments with *Locusta migratoria* showed that the responses of mouthpart palps and antennae to odorants are quite different. Only a few odorants, such as (E,E)-2,4-hexadienal and (E,E)-2,4-heptadienal, elicited stronger electrophysiological responses of both maxillary and labial palps in comparison to the antennae. Additionally, we obtained 114 and 11 putative odorant receptor (OR) gene segments from the antennal and palp transcriptomes, respectively. Two novel odorant-binding proteins (OBPs; *OBP15* and *OBP16*) and one novel OR (*OR142*) were identified for the first time. Out of the 16 OBP genes tested in RT-PCR and qPCR analyses, *OBP8* was highly expressed in the nymphal palps. *OBP4, OBP10*, and *OBP16* were only detected in the antennae. The other 11 OBP genes were jointly expressed in both antennae and palps. The relative expression level of *OBP6* in male palps was much higher than that of female palps. Furthermore, for the 11 OR genes identified in palp transcriptome, the expression levels of *OR12, OR13, OR14*, and *OR18* in the palps were significantly higher than those in the antennae. The *OR12* in palps was demonstrated to be involved in detection of hexanal and E-2-hexenal, as well as (E,E)-2,4-heptadienal. Our results provide information on the different olfactory roles of locust antennae and palps at the molecular level.

## Introduction

Mammals and insects have evolved sophisticated olfactory organs to receive a wide range of chemical stimuli. This distinguished ability enables them to detect and discriminate thousands of odor molecules. Many evidences propose that the different olfactory organs of a species play different roles (Smith, 2007; Su et al., 2009). For many insects, the antennae and mouthpart palps are important olfactory organs (de Bruyne et al., 1999, 2001). Both of them are covered with a variety of chemosensory hairs that house the specialized olfactory sensory neurons (OSNs). The initial step in olfaction involves the binding of hydrophobic odorous molecules to odorant receptors (ORs) located at the ciliated dendrite endings of OSNs (Touhara and Vosshall, 2009). In this process, the high concentration of odorant-binding proteins (OBPs), which liaise the external environment and the ORs, is regarded as the important components in odor transmissions (Pelosi et al., 2006). Therefore, exploration the expressional patterns of olfactory genes in insects antennae and mouthpart palps is the basis for understanding the different roles of these olfactory organs.

Since the first insect OR and OBP were identified in *Drosophila* (Clyne et al., 1999; Gao and Chess, 1999; Vosshall et al., 1999) and in *Antheraea polyphemus* (Vogt and Riddiford, 1981), respectively, several of them have been identified in other insect species (Clyne et al., 1999; Vosshall et al., 1999; Xu et al., 2003; Pelosi et al., 2006; Zhou et al., 2010; Leal, 2013). In the genomes characterized to date, 79 OR genes have been found in mosquito (Hill et al., 2002), 162 in honey bee (Robertson and Wanner, 2006), and 341 in red floor beetle (Engsontia et al., 2008).

Locust (*Locusta migratoria*) is a model animal of hemimetabolous insects, and is also a notorious pest that damage worldwide agricultural productions (Hassanali et al., 2005). The feeding behavior of locust is probably mediated by chemoreception. Currently, 142 OR genes and 14 OBP genes have been identified in its genome and transcriptome^1^ (Ban et al., 2003; Jin et al., 2005; Yu et al., 2009; Wang et al., 2015).

The relatively simple structure of the mouthpart palp represents an attractive model for investigating the neuromolecular networks which underlie chemosensation of an insect (Bohbot et al., 2014). However, the research on the olfactory genes expressed at the palps is limited (de Bruyne et al., 1999; Lu et al., 2007; Sparks et al., 2014; Dweck et al., 2016). For locust, only one study has announced that its antennae and mouthpart palps are responsible for different olfactory functions (Zhang et al., 2017). Here, we used *L. migratoria* as a model to investigate the electrophysiological responses of the palps and antennae as well as the different expression patterns of *OBPs* and *ORs* between these two olfactory organs. The aims of this study are to explore the different physiological functions and molecular bases in olfaction between locust antennae and palps.

## Materials and Methods

### Ethics Statement

All of our experimental materials and methods are not contrary to ethics.

### Insects and Tissues

*Locusta migratoria* individuals were obtained from the Department of Entomology, China Agricultural University. Detailed rearing procedures and tissue extraction were described in Xu et al. (2013).

### Electrophysiological Studies

All electrophysiological experiments were conducted with a 10× universal AC/DC amplifier (Syntech, Netherlands), and the signals were recorded in an Intelligent Data Acquisition Controller (IDAC-4, Syntech, the Netherlands). EagPro software was used to record the absolute amplitudes after stimulation. The experimental chemicals were originally selected from leaf volatiles of maize, wheat, cotton, and soybean (Buttery and Ling, 1984; Buttery et al., 1985; Zeringue and McCormick, 1989; Njagi and Torto, 1996; Shibamoto et al., 2007; Pan et al., 2010; Michereff et al., 2011; Fu et al., 2014; Leppik and Frérot, 2014). Totally, 47 compounds (odorants) with the highest grade available (90–99.9%; Sigma-Aldrich, Shanghai, China) were used in the experiment (**Supplementary Table S1**).

Electrophysiological technique and protocols were followed by the practical introductions of Syntech (2009). For recording electroantennograms (EAGs), the antennae of fifth-instar nymphs were removed from the head and the distal tips of the antennae were immediately cut off. Each antenna was placed between the reference electrode (basal tip) and the recording electrode (distal tip) which connected by Spectra 360 electrode gel. The recordings were proceed since the signal input was stable. Diluted volatile compounds (each 10 μl) were applied to filter paper strips (length 2 cm, width 0.5 cm) which inserted into Pasteur tubes. Each Pasteur tube was only used for testing a specific compound. Paraffin oil was used as a blank control. The tube carried a constant airflow (150 ml/min), and its opening was positioned 1 cm from the antenna. The odor airflow was controlled by a stimulus air controller (CS-55, Syntech, Netherlands) and directed to the surface of the antenna. In this way, the stimuli were provided as 1 s at 20 ml/min generated by the stimulus air controller (CS-55, Syntech, Netherlands). There was an interval of 2 min between two stimulations to enable the recovery of antenna activity. The test was in the following order: paraffin oil (blank control), 20% (v/v) hexanal (positive control), 1% (v/v) chemical (test odorant), and paraffin oil (blank control). Each chemical compound was tested at least three times with different antennae. For electropalpograms (EPGs) recording, the abdomen of the locust was covered with a half-dissected centrifuge tube (0.5 ml), then fixed laterally on a glass slide using sticky tape. The dome of the fixed maxillary or labial palp (with dental wax) was directly oriented to the stimulus-supplying air tube. The reference electrode was inserted into the neck, and the recording electrode was inserted into the basal part of the dome by using an MN-151 micromanipulator (Narishige, Japan). The method was referred to electroantennograms on *Drosophila* (Ayer and Carlson, 1991) and flesh fly, *Neobellieria bullata* (Diptera: Sarcophagidae) (Wasserman and Itagaki, 2003). Each odorant was tested on at least four palps.

The mean value of the EAG, maxillary electropalpogram (EPG-M), or labial electropalpogram (EPG-L) was calculated with the following equation according to Shi et al. (2003):

$$\begin{array}{c} {\mathrm{RV}}_{\mathrm{EAG}}({\mathrm{RV}}_{\mathrm{EPG}})\;=\frac{Vs-Vb}{Vp-Vb} \end{array}$$

where RV_EAG,_ RV_EPG-M_, or RV_EPG-L_ represents the relative value of the response of the relevant receptor, Vs represents the recorded value of the response of the receptor to odorant, Vp represents the recorded value of response of the receptor to the positive control, and Vb represents the recorded value of response of the receptor to the blank control.

### Transcriptome Sequencing

To understand the molecular basis of olfaction in *L. migratoria* antennae and palps, transcriptome sequencing of each organ was performed as previously described by Zhang et al. (2015). In brief, the antennae or a mix of maxillary and labial palps from 30 fifth-instar locust nymphs (aged 3–5 days) were collected and their total RNA was extracted with TRIzol^®^ Reagent (Life Technologies, United States) based on standard protocols. The RNA sample was purified, tested for purity and integrity, and finally introduced into the Illumina HiSeq^®^ 2500 platform (Illumina, San Diego, CA, United States) for sequencing.

The method of *de novo* assembly was originally described by Zhang et al. (2015). In brief, *de novo* assembly of the short reads was performed using SOAPdenovo (Xie et al., 2014) at default parameters. The generated unigenes were analyzed by searching the non-redundant (NR). Unigene analyses were performed on a high-performance server, using a method similar to that originally described by Zhang et al. (2015). In brief, unigenes were annotated and aligned with protein databases from the National Center for Biotechnology Information (NCBI) and Swiss-Prot^2^. The targeted putative OR and OBP genes were then identified. A customized gene identification procedure was undertaken as follows: a local BLAST program, BioEdit (Vision 7.0.4.1) (Hall, 1999) Sequence Alignment Editor, was employed to search for more olfactory genes within the assembled and annotated unigenes library by querying for each of the annotated olfactory unigenes. Parameters were set as follows: minimum identity >95%, length >200 bp and *E*-value < 10^-10^. Finally, all repeatedly aligned olfactory unigenes were removed until only one remained. All single olfactory unigenes were subjected to BLAST alignment in the NCBI online server, and both ends of each unigene open reading frame structure were predicted.

Next, we screened the unigene sequences against protein databases Swiss-prot^2^, COG^3^, and KEGG^4^ with blastx. We used “OR” and “OBP” as keywords to screen the annotated sequences. In order to promote identification of putative target genes, we used the known OBP and OR sequences of *L. migratoria* as “queries” to screen the transcriptome databases with tblastn. The putative OBP and OR genes were then confirmed using blastx. The TMHMM program (v. 2.0)^5^ was used to predict the transmembrane domains of the OR genes.

### Tissue Expression Analysis of OBP and OR Genes

The assay included identification of gene expression in antennae, mouthparts, and guts of fifth-instar nymphs, and antennae and palps of adults of both gender. The tissue expressions of the candidate OBP and OR genes (accession numbers and gene names are listed in **Supplementary Tables S2, S3**) were analyzed with a method similar to that described by Zhang et al. (2015). In brief, total RNA was extracted from the above tissues with TRIzol (Invitrogen, CA, United States). Then, first-strand cDNA was synthesized using the cDNA FastQuant RT Kit (with gDNase) (Tiangen Biotech Co. Ltd., Beijing, China). The PCR product was sequenced to verify the specificity of primers used in RT-PCR. These gene-specific primers of OBP and OR were designed with Primer-BLAST (**Supplementary Tables S2, S3**). qPCR assays were performed in the StepOnePlus^TM^ Real-time PCR System (Applied Biosystems, United States), with a KAPA SYBR^®^ FAST qPCR Kit Master Mix (2X) (KAPA Biosystems, Boston, MA, United States). A qRT-PCR assay is similar to Zhang et al. (2015). In brief, the assay was carried out in a 20 μl reaction mixture in the ABI 7900 system (Applied Biosystems, Carlsbad, CA, United States). PCR was performed under the following program: 95°C for 3 min, 40 cycles at 94°C for 15 s, 60°C for 20 s, and extension at 72°C for 15 s. The melting curve was analyzed to assure specificity of the primers after each reaction and the 2^-ΔΔCT^ method (Livak and Schmittgen, 2001) was used to calculate the expression level of each OBP and OR gene. Each sample type was replicated three times. The differences between relative expression levels of OBP or OR genes were analyzed with *t*-tests. The β-actin was used as a reference gene for internal standardization. PCR efficiency and specificity of primers to the target genes were validated in the experiment.

### Phylogenetic Analysis of OBPs of *L. migratoria* and Other Insects

We constructed a phylogenetic tree using the 16 candidate OBPs of *L. migratoria* and selected OBPs of other insects, including *Oedaleus asiaticus, Drosophila melanogaster, Bombyx mori*; *Tribolium castaneum*; *Adelphocoris lineolatus, Apis mellifera* (the OBP amino acid sequences of all OBPs in this experiment are listed in **Supplementary Table S5**). We renamed *Lmig*OBP13 (OBP4, GenBank: AEX33160.1,) and *Lmig*OBP14 (OBP5, GenBank: AEX33161.1), on account *Lmig*OBP4 and *Lmig*OBP5 have been registered previously with the number AEV45802.1 and AFL03411.1 in NCBI GenBank by our lab. The phylogenetic tree was constructed by the neighbor-joining method with Poisson-modified distance with MEGA6 software.

### RNA Interference

Double stranded RNA (dsRNA) was synthesized based on manufacturer manual. In brief, PCR products were amplified with T7 promoter conjugated primer (primer pairs see **Supplementary Table S6**), and then purified with Wizard^®^ SV Gel and PCR Clean-Up System (Promega, United States) as templates for *in vitro* transcription. dsRNA was synthesized with T7 RiboMAX^TM^ Express RNAi System (Promega, United States) and diluted into 1000 ng/μl with ddH_2_O and stored at -20°C. Target dsRNA (5 μg) was delivered into each locust dorsal vessel through inter-segmental membrane (1st day of 5th instar nymph) by IM-9B microinjector (Narishige, Japan) equipped with glass capillary. dsGFP was microinjected as control group. The treated locusts were normally raised as wild individuals. RNA silencing was checked between 3th and 5th day post-injection. All RNAi-treated locusts used in EAG or EPG were checked by PCR after electrophysiological experiment to confirm the results of silencing. EAG or EPG methods are similar to those described above. The response value of the EAG or maxillary electropalpogram (EPG-M) was calculated with the following equation: RV_EAG_ (RV_EPG_) = Vs - Vb. Where RV_EAG_ or RV_EPG_ represents the value of the response of the relevant receptor, Vs represents the recorded value of the response of the receptor to odorant, and Vb represents the recorded value of response of the receptor to the blank control. Each chemical compound was tested on at least seven different antennae or maxillary palps.

### Statistical Analysis

Electroantennograms and EPG results were compared with one-way ANOVA with *post hoc t*-tests. All data was analyzed using GraphPad Prism 7 (Graphpad software, San Diego, CA, United States).

## Results

### Different Electrophysiological Responses of Locust Antennae and Palps to the Odors

**FIGURE 1:**
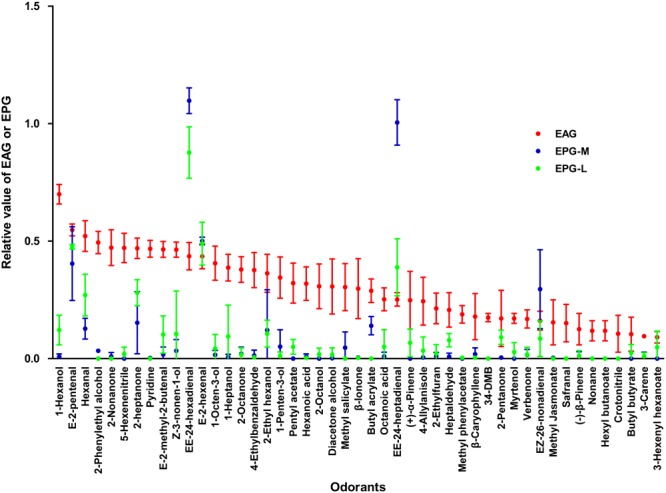
Electrophysiological responses of *Locusta migratoria* antennae, and maxillary and labial palps to odorants from plants. EAG, electroantennogram; EPG-M, electropalpogram of maxillary palps; EPG-L, electropalpogram of labial palps. Sample size for each odorant, *n* = 3–6 at 1% v/v concentration. The bars indicate standard errors of the mean.

Locust antennae, maxillary palps and labial palps showed responses to most of the 47 tested odorants at 1% v/v. However, the relative electrophysiological responses of the antennae were stronger than ttronger than those of the palps to 43 out of 47 odorants. Of the 43 odorants, 19 of them elicited responses only in the antennae. Contrasted to the antennae could be elicited strong responses by a plenty of odorants, only two odorants, (E,E)-2,4-hexadienal and (E,E)-2,4-heptadienal, induced stronger electrophysiological responses to both the maxillary and labial palps than to the antennae (**Figure 1**). Therefore, locust antennae and palps perceive odorants differently to some extent.

### Different Expression of OBP in Locust Antennae and Palps

**FIGURE 2:**
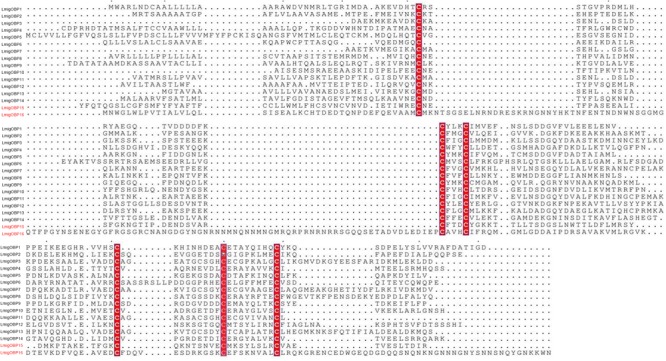
Alignment of amino acid sequences of two novel odorant binding proteins (OBPs) and 14 other OBPs of *L. migratoria*. The novel odorant binding proteins are highlighted in red. Conserved cysteines are within the red boxes.

From our analysis of the transcriptomes of locust antennae and palps, two novel OBPs, named as *Lmig*OBP15 and *Lmig*OBP16 were identified. Together with the previously annotated 14 OBPs^6^ (Ban et al., 2003; Jin et al., 2005; Yu et al., 2009;), a total of 16 OBPs were obtained in transcriptomes. All of them were closest to the OBPs from another locust, *O. asiaticus* (Zhang et al., 2015), in the phylogenetic tree (**Supplementary Figure S1**). Among the 16 OBPs, the longest amino acid sequence was OBP16, with 271 amino acids; while the shortest was OBP7, with only 133 amino acids. OBP3, OBP7, OBP11, and OBP13 were “Plus-C” OBPs (Zhou et al., 2004) (**Figure 2** and **Supplementary Table S5**). Sequence identities of the 16 OBPs ranged from 9.2 to 60.0% (**Table 1**).

**Table 1:** Consensus alignment (%) of 16 odorant binding protein (OBP) amino acid sequences of *L. migratoria*.

	OBP1	OBP2	OBP3	OBP4	OBP5	OBP6	OBP7	OBP8	OBP9	OBP10	OBP11P11	OBP12	OBP13	OBP14	OBP15
OBP2	16.2														
OBP3	15.1	19.0													
OBP4	14.7	16.2	16.5												
OBP5	30.7	20.7	21.1	16.7											
OBP6	13.0	13.2	14.0	12.9	11.4										
OBP7	17.2	18.7	42.9	18.6	11.6	17.7									
OBP8	15.7	18.3	15.2	22.7	19.3	17.9	17.2								
OBP9	25.2	16.2	16.7	11.8	22.1	12.4	16.2	16.2							
OBP10	14.8	18.8	20.9	37.9	16.7	14.6	19.5	22.3	15.0						
OBP11	15.7	14.6	48.9	20.5	13.5	14.6	60.0	12.6	15.5	22.2					
OBP12	17.2	15.6	14.3	20.5	19.3	9.2	13.2	14.5	13.6	18.4	14.8				
OBP13	15.6	17.1	42.1	17.8	17.9	10.5	41.9	17.2	16.2	16.9	39.3	15.5			
OBP14	11.2	19.5	18.8	35.5	14.9	12.7	14.2	21.8	16.0	32.3	15.1	20.2	20.0		
OBP15	12.6	11.8	14.5	20.9	15.3	13.0	14.5	17.7	10.8	21.7	15.5	28.7	18.2	22.6	
OBP16	16.8	12.1	12.2	12.1	16.7	12.5	12.0	11.9	11.6	14.3	12.9	11.0	9.8	10.4	11.3

*Values in red boxes indicate the highest and the lowest identity of the OBPs.*

RT-PCR analyses for OBPs showed that *OBP4, OBP10*, and *OBP16* were only expressed in the antennae of nymphs and female and male adults. Expression level of *OBP8* was higher in larval palps than that in adult palps and other tested organs in both adult and nymph. Additionally, *OBP1, OBP2, OBP3, OBP5, OBP6, OBP11, OBP12, OBP13*, and *OBP14* were expressed in the antennae, palps, and mid gut (**Supplementary Figure S2** and **Supplementary Presentation S1**).

**FIGURE 3:**
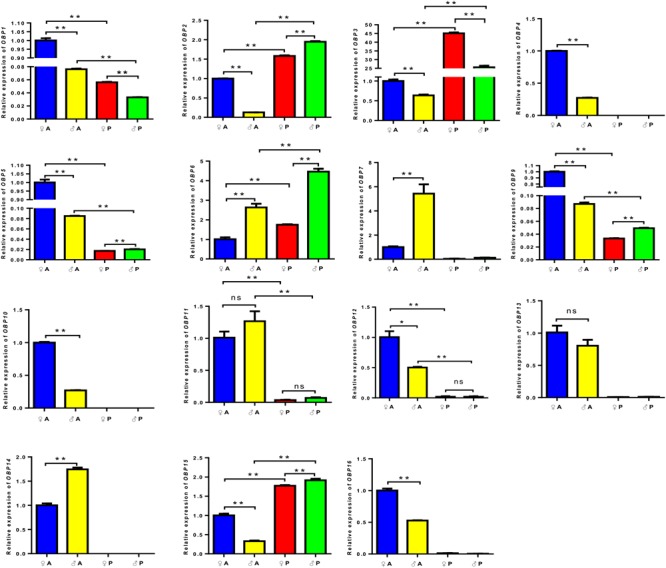
Comparison of relative quantitative levels of expression of 15 odorant binding protein (OBP) genes in the antennae and palps of *L. migratoria* females (♀) and males (♂) by qRT-PCR. A, antennae; P, maxillary and labial palps; ^∗^, significant difference at *p* < 0.05 level (*t*-text); ^∗∗^, significant difference at *p* < 0.01 level (*t*-test). The bars indicate standard errors of the mean for three independent experiments.

Our qPCR results revealed the relative expression levels of 15 OBP genes in the chemosensory organs, except for *OBP8*, which was too difficult to be detected in adult antennae and palps (**Figure 3** and **Supplementary Table S4**). The expression levels of 11 OBP genes, including *OBP1, OBP4, OBP5, OBP7, OBP9, OBP10, OBP11, OBP12, OBP13, OBP14*, and *OBP16*, in the antennae were significantly higher than those in the palps of the same sexual individuals. In contrast, *OBP2, OBP3, OBP6*, and *OBP15* were markedly up-regulated in the palps than those in the antennae of both genders. Interestingly, the relative expression level of *OBP3* in female palps was much higher than that in male palps yet in the antennae of both sexes.

Expression levels of 10 OBP genes in female antennae, including *OBP1, OBP2, OBP3, OBP4, OBP5, OBP9, OBP10, OBP12, OBP15*, and *OBP16*, were significantly higher than those in male antennae. On the other hand, expression levels of *OBP6, OBP7*, and *OBP14* in male antennae were significantly higher than those in female antennae. The expressions of *OBP1* and *OBP3* in female palps were higher than those in male palps, whereas *OBP11* and *OBP12* were expressed at similar levels in the palps of both sexes. *OBP2, OBP5, OBP6, OBP9*, and *OBP15* in male palps were highly expressed than in female palps (**Figure 3** and **Supplementary Table S4**).

### Different Expression of Odorant Receptors in the Antennae and Palps

**FIGURE 4:**
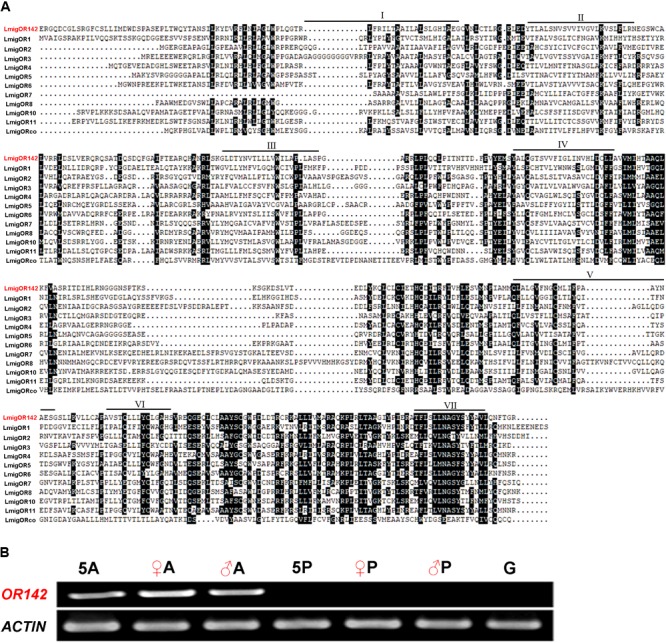
Alignment of amino acid sequences of a novel odorant receptor (OR) and 11 other ORs of *L. migratoria* **(A)**, and expression of the novel OR gene in tissues **(B)**. The novel odorant receptor is highlighted in red. The amino acid in back boxes, over 50% similarity; black bars with Roman numerals, the predicted transmembrane domains; ♀, female; ♂, male; A, adult antennae; P, adult palps; G, fifth-instar gut; 5A, fifth-instar antennae; 5P, fifth-instar maxillary and labial palps; Actin, actin gene as positive control.

We identified 114 putative OR gene segments (35 putative OR genes with more than 300 amino acids) from the transcriptome of locust antennae. However, only 11 putative OR gene segments were identified from the transcriptome of palps. Notably, *OR142* from the antennal transcriptome was identified for the first time. It has 408 amino acid residues with 7 predicted transmembrane domains (**Figure 4A**). RT-PCR also showed that this gene was only expressed in the antennae (**Figure 4B**).

**FIGURE 5:**
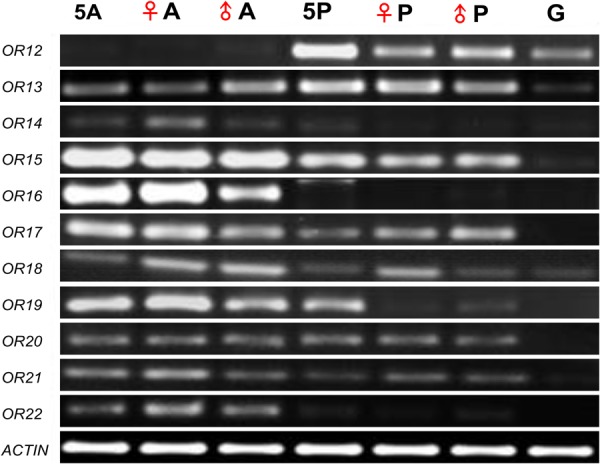
Expression of 11 odorant receptor (OR) genes identified from palp transcriptomes in different tissues of *L. migratoria* by RT-PCR. ♀, female; ♂, male; A, adult antennae; P, adult palps; G, adult gut; 5A, fifth-instar antennae; 5P, fifth-instar maxillary and labial palps; Actin, actin gene.

We checked the expressions of 11 putative OR genes identified from the palps using RT-PCR (**Figure 5**). Interestingly, only the *OR12* was not detected in the antennae. *OR16* was only detected in the antennae. *OR13, OR15, OR18*, and *OR21* were widely expressed in the antennae, palps, and gut of nymphs and adults of both sexes. *OR14, OR17, OR19, OR20*, and *OR22* were jointly expressed in the antennae and palps of nymphs and adults of both gender.

**FIGURE 6:**
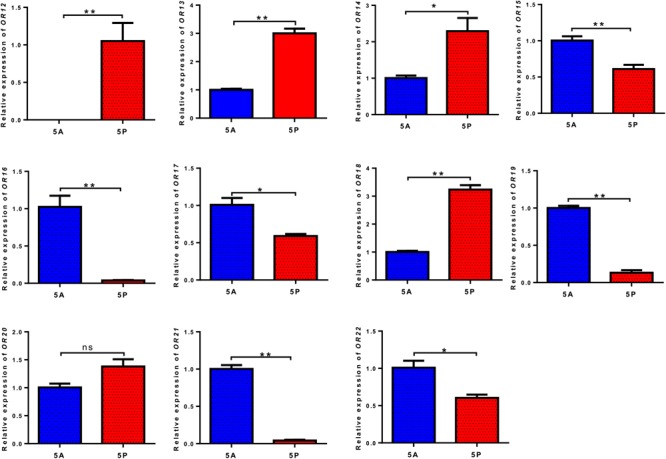
Quantitative levels of expression of 11 odorant receptors (ORs) in the palps relative to their levels of expression in the antennae of fifth-instar nymphs of *L. migratoria* by qRT-PCR. 5A, antennae; 5P, palps; ^∗^, significant difference at *p* < 0.05 (*t*-test); ^∗∗^, significant difference at *p* < 0.01 (*t*-test). The bars indicate standard errors of the mean for three independent experiments.

In the fifth-instar nymph, the relative expression levels of *OR12, OR13, OR14*, and *OR18* genes in the palps were significantly higher than those in the antennae. In contrast, expression levels of *OR15, OR16, OR17, OR19, OR21*, and *OR22* in the antennae were significantly higher than those in the palps (**Figure 6**). Expression levels of *OR20* did not show significant differences between the antennae and the palps (**Supplementary Table S4**).

### An Odorant Receptor Specifically Expressed in Palps Was Involved in Detection of Three Aldehydes

**FIGURE 7:**
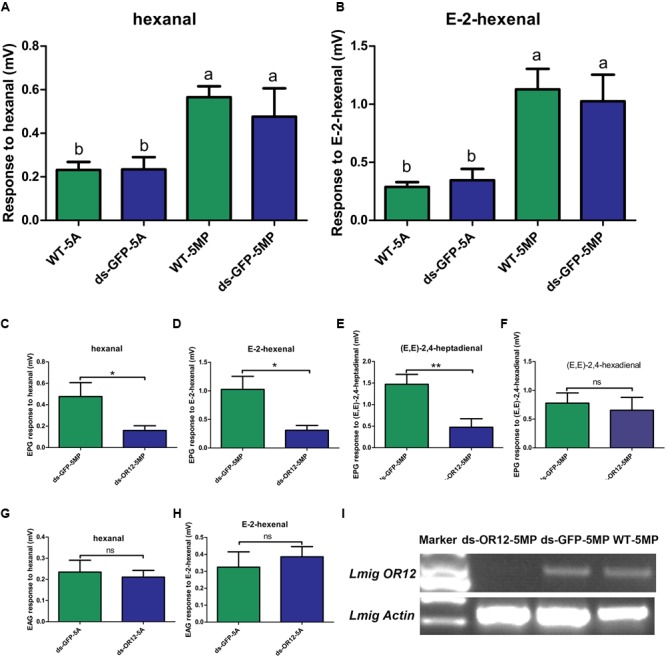
An odorant receptor specifically expressed in palps involved in detection of aldehydes. **(A)** Comparison of response level to hexanal in different organs and different genotypes by EPG or EAG. Abbreviations: 5A, the antenna of 5th instar nymph; 5MP, the maxillary palp of 5th instar nymph; WT, wild type; ds-GFP, dsRNA of GFP injected. The response of EPG or EAG was calculated from the response value of maxillary palp or antenna treated with chemicals minus the response value of maxillary palp or antenna treated with mineral oil as control. Error bar indicates SEM. ^∗^*p* < 0. 05, one-way ANOVA with *post hoc t*-tests. **(B)** Comparison of response level to E-2-hexenal in different organs and different genotypes by EPG or EAG. Abbreviations are referred to **(A)**. **(C)** Comparison of response level to hexanal in different genotypes by EPG. Abbreviations are referred to **(A)**. ds-OR12, dsRNA of OR12 injected. **(D)** Comparison of response level to E-2-hexenal in different genotypes by EPG. Abbreviations are referred to **(A)**. ds-OR12, dsRNA of OR12 injected. **(E)** Comparison of response level to (E,E)-2,4-heptadienal in different genotypes by EPG. **(F)** Comparison of response level to (E,E)-2,4-hexadienal in different genotypes by EPG. **(G,H)** Comparison of response level to hexanal or E-2-hexenal in different genotypes by EAG. **(I)** RNA silencing is checked after electrophysiological experiment with semi-quantitative RT-PCR. Actin was used to check template quality.

Our electrophysiological experiments showed that the palps responded remarkably stronger to (E,E)-2,4-heptadienal and (E,E)-2,4-hexadienal than antennae (**Figure 1**). Besides, we also found that another two odorants, hexanal and E-2-hexenal, elicited stronger absolute values in EPG than in EAG (**Figures 7A,B**). We speculated that there would be some specific ORs expressed in palps, which are responsible for the detection of these chemicals. Meanwhile, the RT-PCR analysis indicated that *OR12* was highly expressed in palps. Thus we presumed that *OR12* might be involved in detection to the aldehydes. We found that the responses of EPG of locust nymphs injected with dsRNA of *OR12* to hexanal and E-2-hexenal were significantly reduced in comparison with locust injected with dsRNA of GFP (**Figures 7C,D**). Interestingly, the response of EPG of locust nymphs injected with dsRNA of OR12 to (E,E)-2,4-heptadienal was significantly lower than that of animals injected with dsRNA of GFP (**Figure 7E**). In turn, no changes of EPGs were detected to (E,E)-2,4-hexadienal between the two dsRNA experimental animals (**Figure 7F**). In contrast, there was no significant difference in EAG responses to hexanal and E-2-hexenal between the *OR12* and GFP dsRNA injected locusts (**Figures 7G,H**). Moreover, the expression level of *OR12* in palps was indeed depressed by injection of dsRNA of *OR12* in comparison with individuals injected with dsRNA of GFP, or wild type (**Figure 7I**).

## Discussion

Locusts antennae has many olfactory sensilla of the basiconic, trichoid, and coeloconic type, while only few basiconic sensilla are present on the dome of each palp (Ochieng and Hansson, 1999; Jin et al., 2006). In the present study, the electrophysiological responses of antennae were stronger than those of palps to most tested odorants. We speculated that the abundant neurons and chemoreception proteins in the antennae, such as ORs and OBPs, induced this result. Since the varieties of odorants tested in this study were limited, we did not screen any odorant which only elicit response to palps. However, four odorants, (E,E)-2,4-hexadienal, (E,E)-2,4-heptadienal, hexanal and E-2-hexenal elicited much stronger responses to palps in comparison to the antennae. This implies that sensilla on the palps may house neurons with special olfactory receptors sensitive to these odorants.

It has been demonstrated that OBPs increase the sensitivity of odor discrimination for insects (Laughlin et al., 2008). The numbers of OBPs vary among insect species (Pelosi et al., 2006). In the present study, we identified two novel OBPs. As a result, there are a total of 16 OBPs found in *L. migratoria*^7^ (Ban et al., 2003; Jin et al., 2005; Yu et al., 2009). Similarly, 15 and 14 OBPs were identified in the antennal transcriptomes of *O. asiaticus* (Zhang et al., 2015) and *Schistocerca gregaria* (Jiang et al., 2017), respectively. Orthopteran insects possess a significantly smaller number of OBPs compared to Dipteran insects, such as *Drosophila* and mosquitoes contain 51 and 79 OBPs, respectively (Galindo and Smith, 2001; Biessmann et al., 2002; Hekmat-Scafe et al., 2002; Xu et al., 2003; Zhou et al., 2004; Hansson and Stensmyr, 2011). This may reflect the specific evolutionary level of locust chemosensory system (Vogt, 2002; Pelosi et al., 2006; Xu et al., 2013).

PCR experiment demonstrated that a greater number of OBPs are expressed in locust antennae than in the palps. This may suggest that the olfactory functions of antennae are different from the palps. However, the relative expression levels of *OBP6* are much higher in male palps than in female palps, indicating that it might be involved in detecting odors from the female. In addition, an extremely high level of *OBP8* expressed in the palps of locust nymphs, suggesting that this protein may be involved in detecting specific odors that are important during nymphal stages. Moreover, the relative lower amounts of olfactory genes in palps may explain why the maxillary palps respond to a narrow range of odors.

Although more than 100 putative OR genes have been identified in the antennae of locust (this study; Wang et al., 2014, 2015), we only identified 11 OR genes in the locust palps. The different OR repertoires imply that the antennae are more versatile in olfaction than the palps. This is similar to the results in *Anopheles gambiae*, where there are more than 60 ORs found in the antennae, but only 13 were found in their palps (Latrou and Biessmann, 2008). Interestingly, our result showed that *OR12* (named *OR6* in Wang et al., 2015) was highly expressed in the palps than antennae of fifth-instar nymphs; but the expressional level of *OR12* in nymphal palps was much lower than that in adult palps. A previous study showed a similar result for this gene in palps of fourth-instar nymphs (Wang et al., 2015). *OR12* may have an important function in the palps at nymphal and adult stages. On the other hand, we found that the *OR14* (named *OR50* in Wang et al., 2015) was weakly expressed in the antennae and palps of both adults and nymphs. Additionally, it was proposed that *OR13* (named *OR133* in Wang et al., 2015) was only expressed in locust antennae, but it was detected in both antennae and palps in the present study. Similarly, *OR17* (named *OR5* in Wang et al., 2015) has previously been detected only in the adult antennae (Wang et al., 2015). However, in our study we detected *OR17* in the antennae of both adults and fifth-instar nymph.

The qPCR data show that the expression of the *OR12, OR13, OR14*, and *OR18* in the palps was significantly higher than in the antennae of fifth-instar nymphs. Contrarily, the expression of *OR15, OR16, OR17, OR19, OR21*, and *OR22* was much higher in the antennae than in the palps. Similar results for OBPs expressed in antennae and palps further suggested that these two chemosensory organs might have different roles in chemoperception. In mosquitoes, the expression level of *AsteOBP1* in antennae was ∼900-fold higher than that in maxillary palps (Sengul and Tu, 2010a,b). Therefore, the presence or absence of OBPs/ORs in the antennae and palps may reflect a natural selection of olfactory traits during the evolution of insect lineages (de Bruyne et al., 1999; Yasukawa et al., 2010).

Our results of RNAi demonstrated that *OR12* in maxillary palps was responsible for detection of hexanal and E-2-hexenal, as well as (E,E)-2,4-heptadienal. This information partially provides a molecular basis for the antenna and palp in different olfactory functions. In *Drosophila*, although the antennae and palps respond to a similar spectrum of odorants, the palps display fewer high-sensitivity responses to specific odorants (Dweck et al., 2016), which also indicates the different roles of their antennae and palps in chemoperception. However, our experiments did not demonstrate that EPG of locust changed to (E,E)-2,4-hexadienal after depression of *OR12*. This odorant might be detected by other ORs, such as *OR13, OR14*, or *OR18*, which were demonstrated to be highly expressed in palps (**Figure 6**). The novel expression of olfactory receptors in the maxillary palps could generate a subpopulation of insects using new food source. In turn, the utilization of new resource, combined with a segregation event, may lead to the emergence of a new species.

In sum, our results show that (E,E)-2,4-hexadienal, (E,E)-2,4-heptadienal, hexanal and E-2-hexenal elicits much stronger responses in palps than in the antennae. We found that *OBP8, OR12, OR13, OR14*, and *OR18* were much higher expressed in the nymphal palps, suggesting that those proteins may be involved in detecting specific odors during feeding process. On the other hand, *OR12* shows specific expression in palps and we showed that it was involved in the detection of three aldehydes produced by the host plant (Buttery and Ling, 1984; Buttery et al., 1985). Consequently, the palps could play an important role in speciation through food selection. The palps, therefore, would be a fruitful area for investigating the specific roles in insect chemoperception in the future.

## Author Contributions

LZ designed the experiments and wrote the manuscript. HL and PW did the electrophysiological experiment. LZ and HL analyzed the transcriptomes and identified the OBP and OR genes. HL, XX, and ZC conducted the PCR experiments. HL, PW, and LZ analyzed the data. All authors contributed to the revisions.

## Conflict of Interest Statement

The authors declare that the research was conducted in the absence of any commercial or financial relationships that could be construed as a potential conflict of interest.

## Footnotes

1 https://www.ncbi.nlm.nih.gov/unigene?term=LOCUST+OBP&cmd=DetailsSearch

2 http://www.uniprot.org/

3 http://www.ncbi.nlm.nih.gov/COG/

4 http://www.genome.jp/kegg/

5 http://www.cbs.dtu.dk/services/TMHMM-2.0/

6 https://www.ncbi.nlm.nih.gov/unigene?term=LOCUST+OBP&cmd=DetailsSearch

7 https://www.ncbi.nlm.nih.gov/unigene?term=LOCUST+OBP&cmd=DetailsSearch

## Supplementary Material

FIGURE S1 Phylogenetic analysis of OBPs of *L. migratoria* and other insects.

FIGURE S2 Tissue expression analysis of OBP genes.

TABLE S1 Details of odorants used in EAG and EPG tests.

TABLE S2 Details of OBPs in *Locusta migratoria* and the primers used for qPCR.

TABLE S3 Details of ORs in *L. migratoria* and the primers used for qPCR.

TABLE S4 The OBP and OR genes result of qPCR.

TABLE S5 List of OBPs of *L. migratoria* and 6 other orders of insects used for the phylogenetic tree.

TABLE S6 Sequence of primers used in RNAi experiments. T7 promoter is shown in red.
