# Supplementary figures and images for: Expressions of Olfactory Proteins in Locust Olfactory Organs and a Palp Odorant Receptor Involved in Plant Aldehydes Detection
Source: Front Physiol. 2018 Jun 4;9:663. doi: 10.3389/fphys.2018.00663 (PMC5994405; doi:10.3389/fphys.2018.00663)

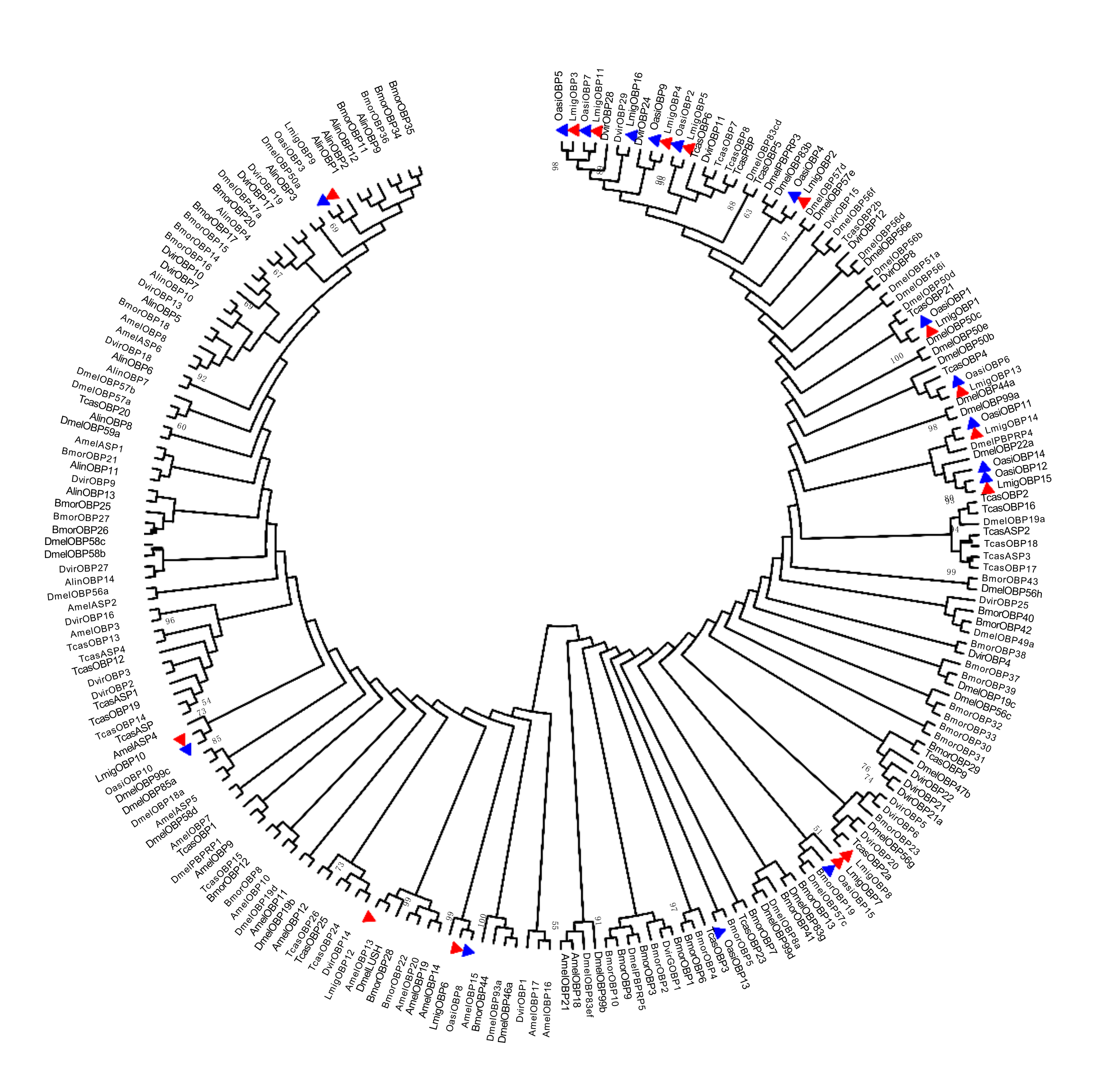

Supplement: FIGURE S1 — Phylogenetic analysis of OBPs of L. migratoria and other insects. [file Image_1.TIF]

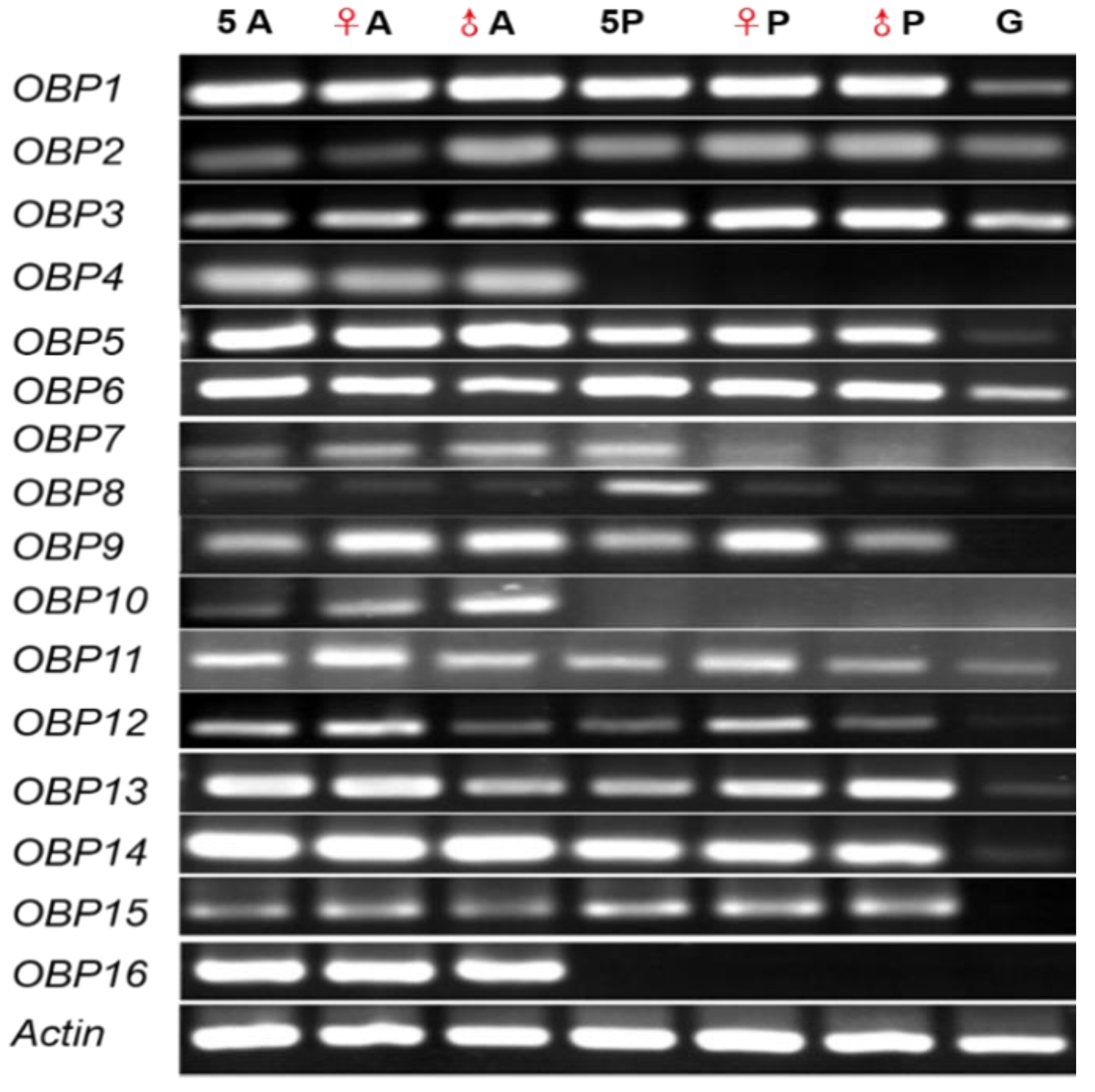

Supplement: FIGURE S2 — Tissue expression analysis of OBP genes. [file Image_2.TIF]

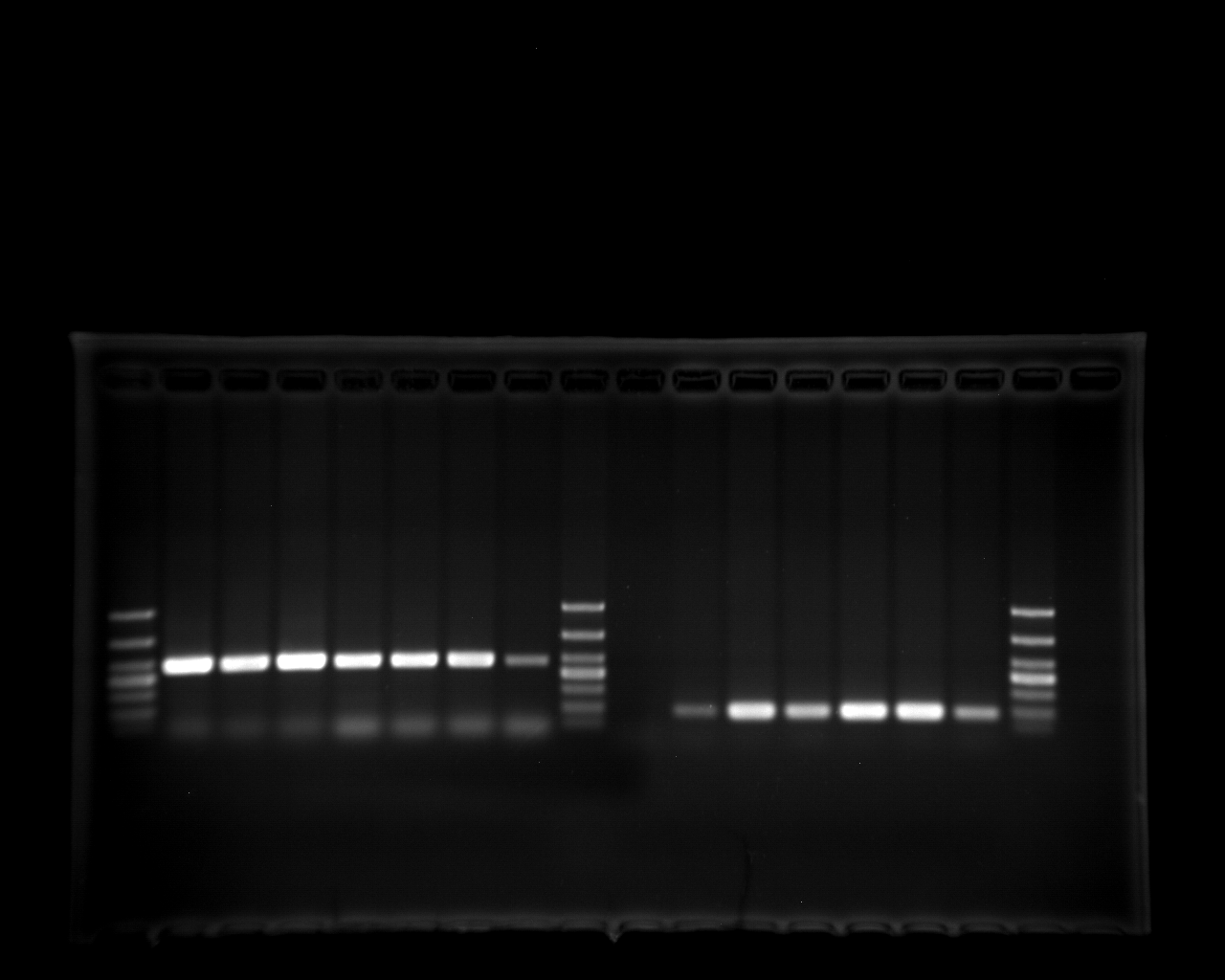

Supplement: Supplementary file 9 [file Presentation_1.ZIP › ╠⌠╤í/OBP1-first group.tif]

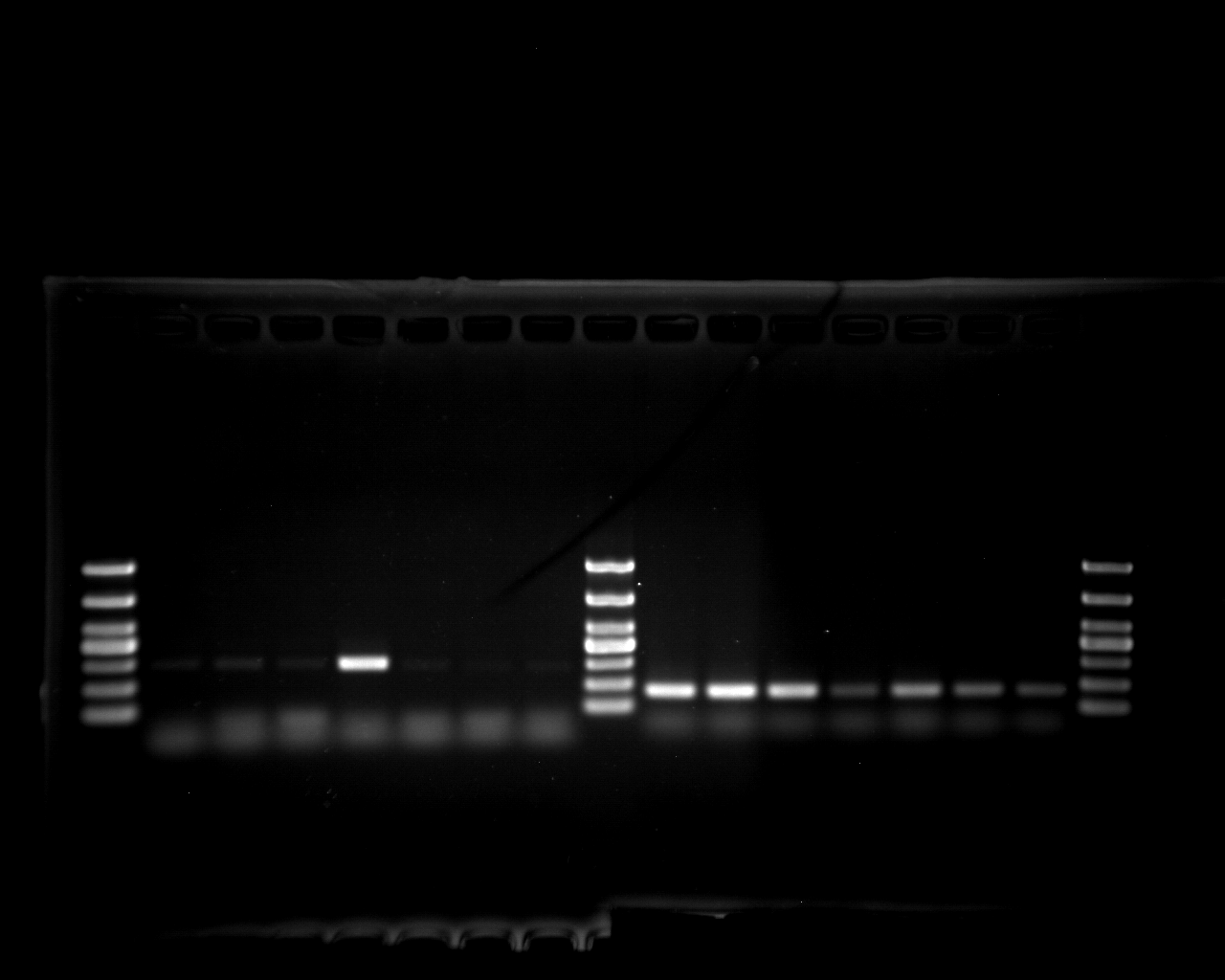

Supplement: Supplementary file 9 [file Presentation_1.ZIP › ╠⌠╤í/OBP10-second group.tif]

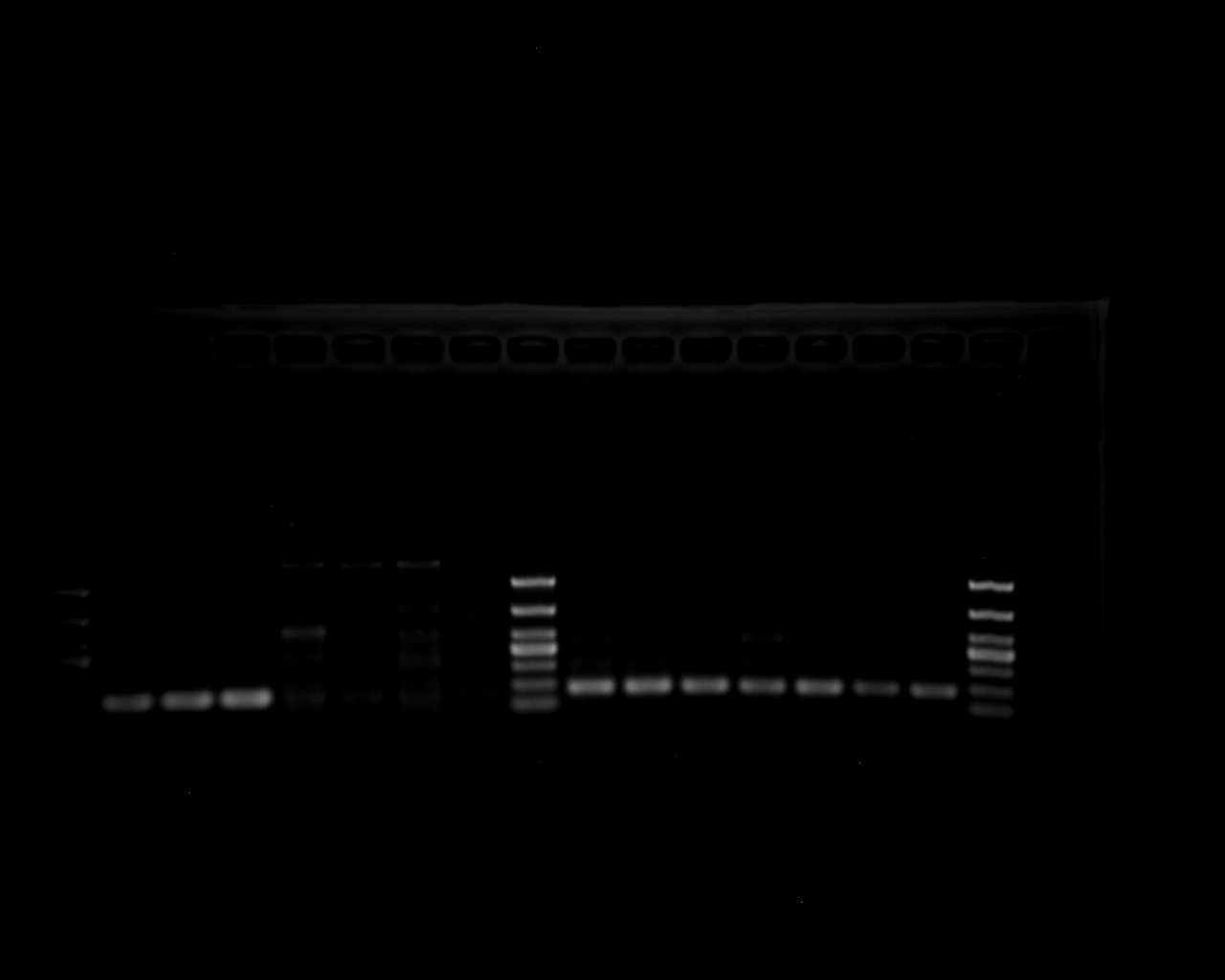

Supplement: Supplementary file 9 [file Presentation_1.ZIP › ╠⌠╤í/OBP11-second group.tif]

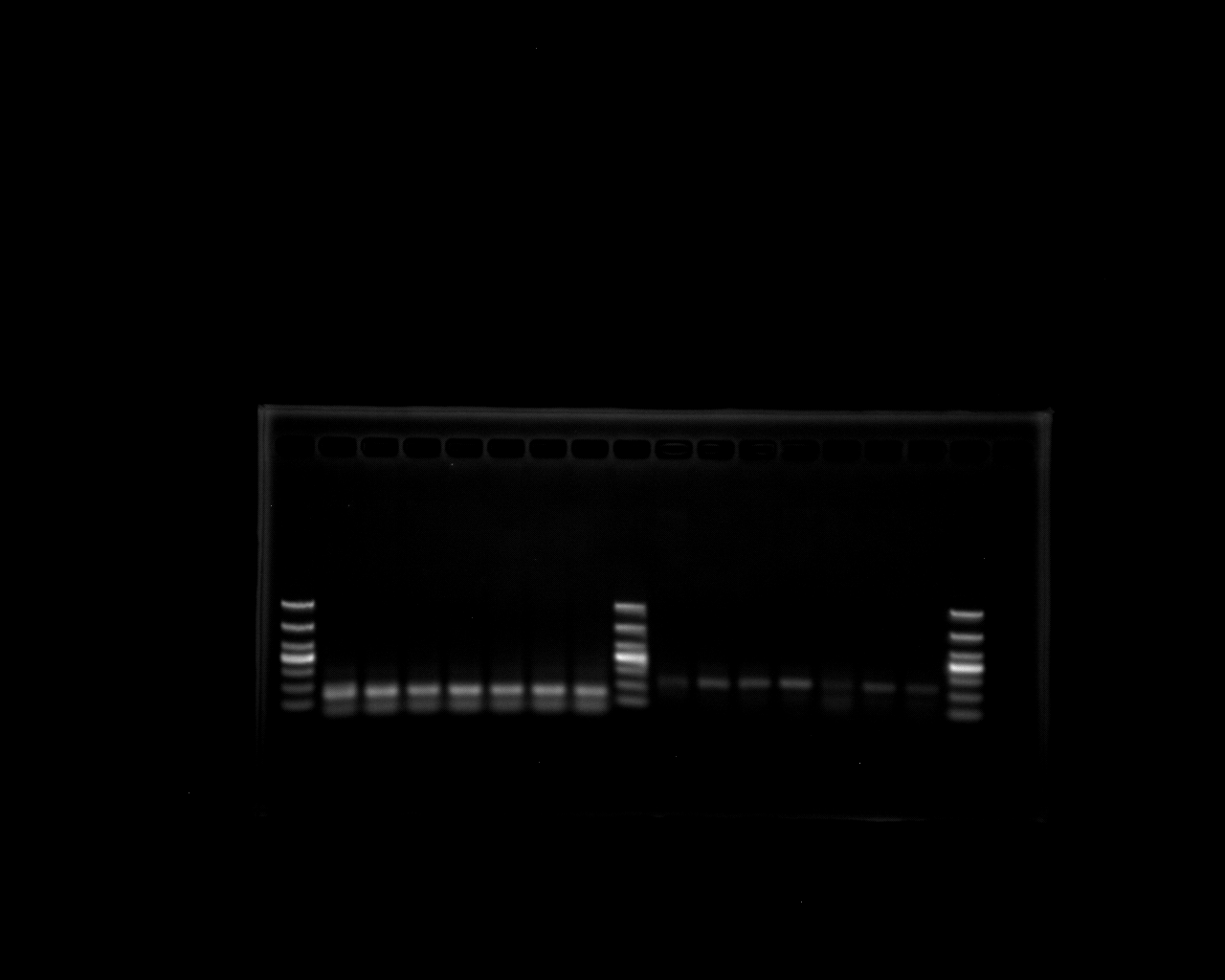

Supplement: Supplementary file 9 [file Presentation_1.ZIP › ╠⌠╤í/OBP12-second group.tif]

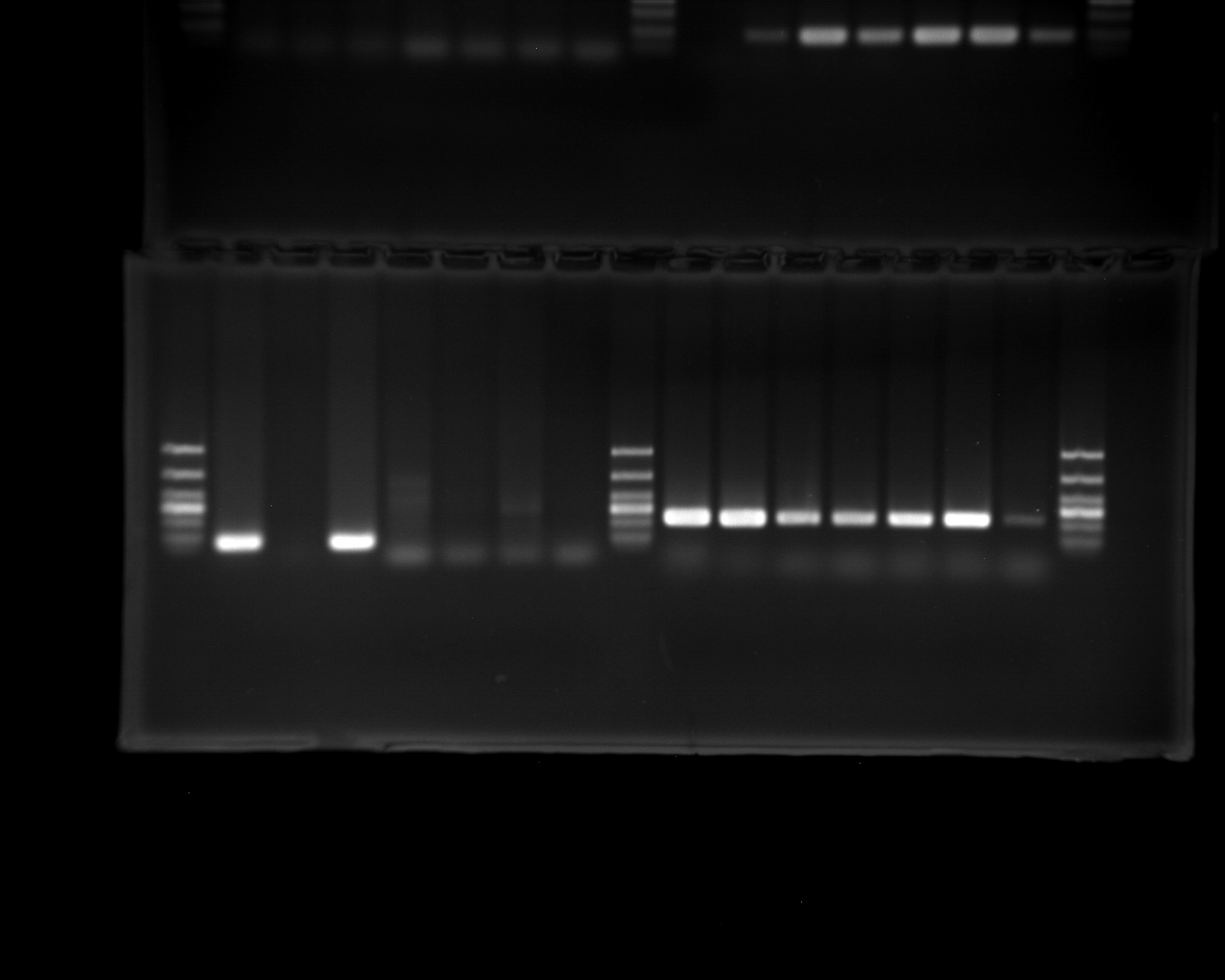

Supplement: Supplementary file 9 [file Presentation_1.ZIP › ╠⌠╤í/OBP13-second group.tif]

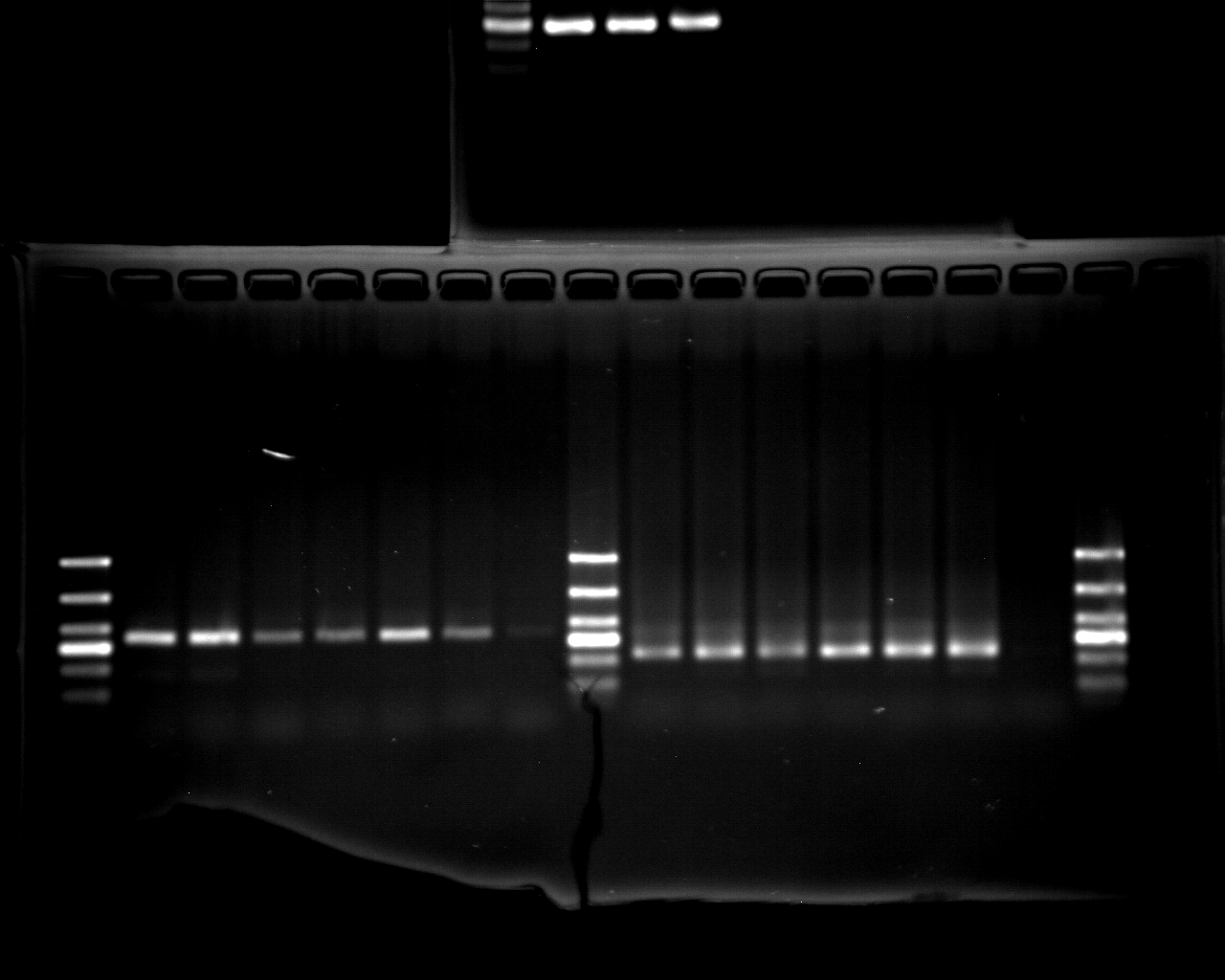

Supplement: Supplementary file 9 [file Presentation_1.ZIP › ╠⌠╤í/OBP15-second group.tif]

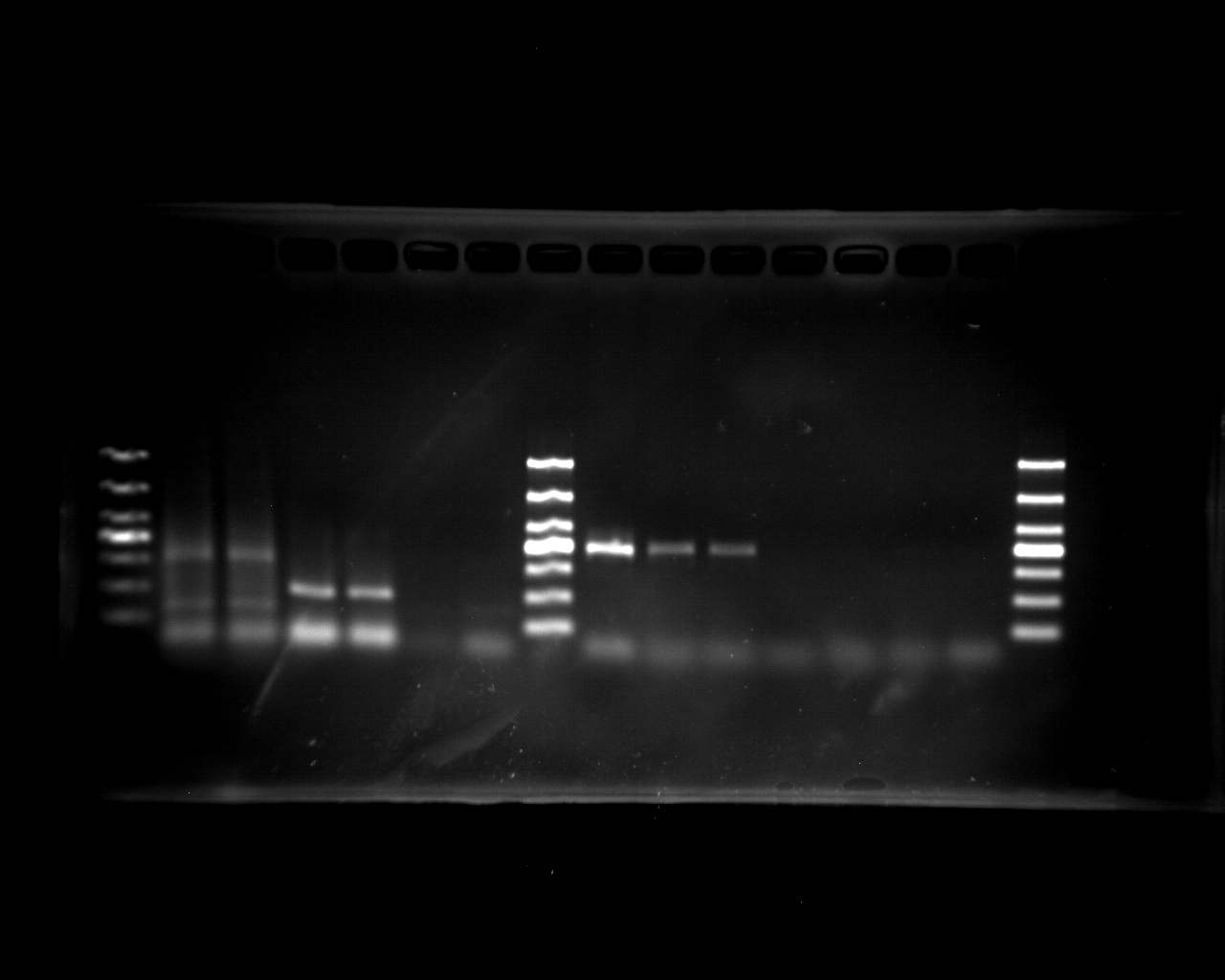

Supplement: Supplementary file 9 [file Presentation_1.ZIP › ╠⌠╤í/OBP16-second group.tif]

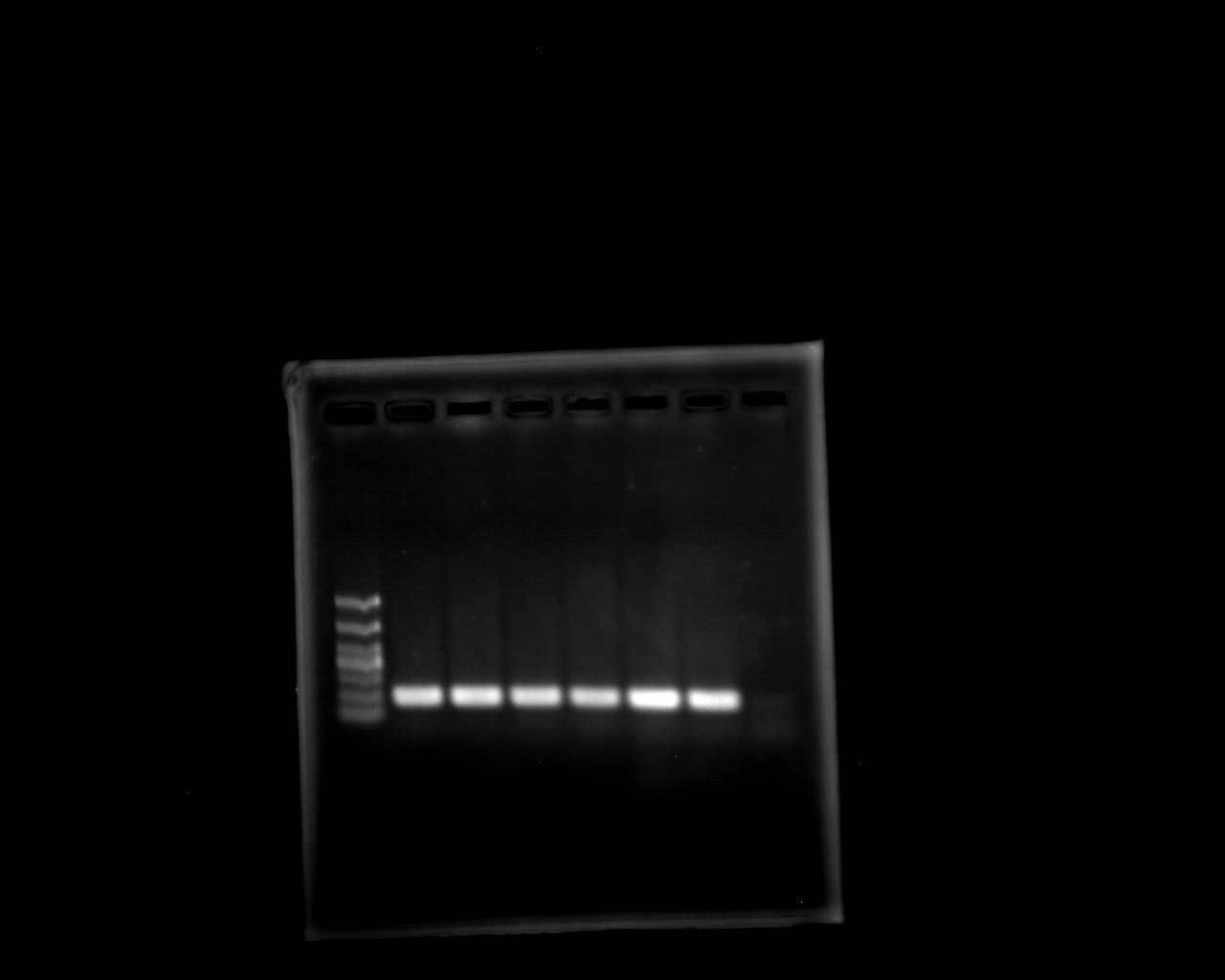

Supplement: Supplementary file 9 [file Presentation_1.ZIP › ╠⌠╤í/OBP2..tif]

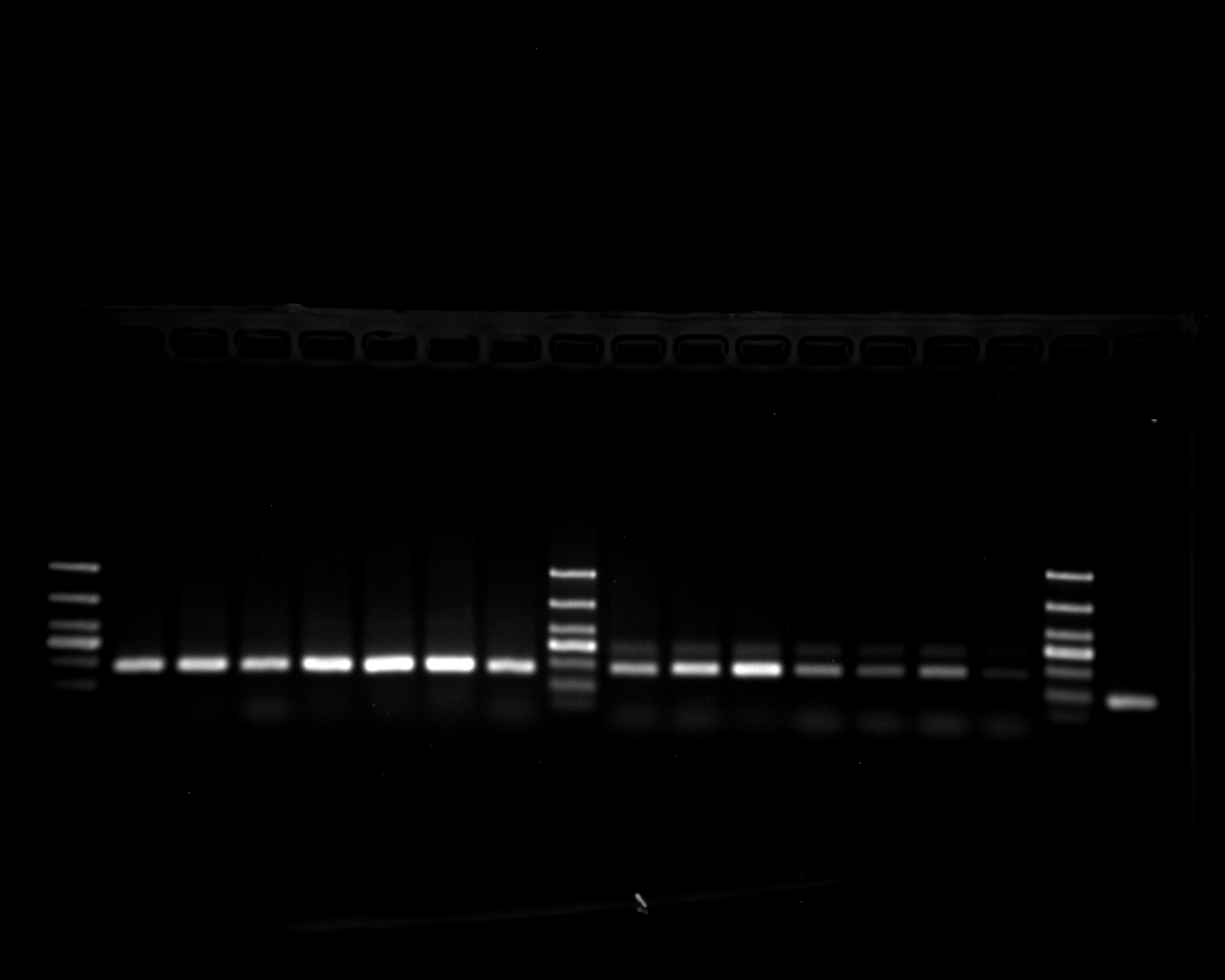

Supplement: Supplementary file 9 [file Presentation_1.ZIP › ╠⌠╤í/OBP3-first group.tif]

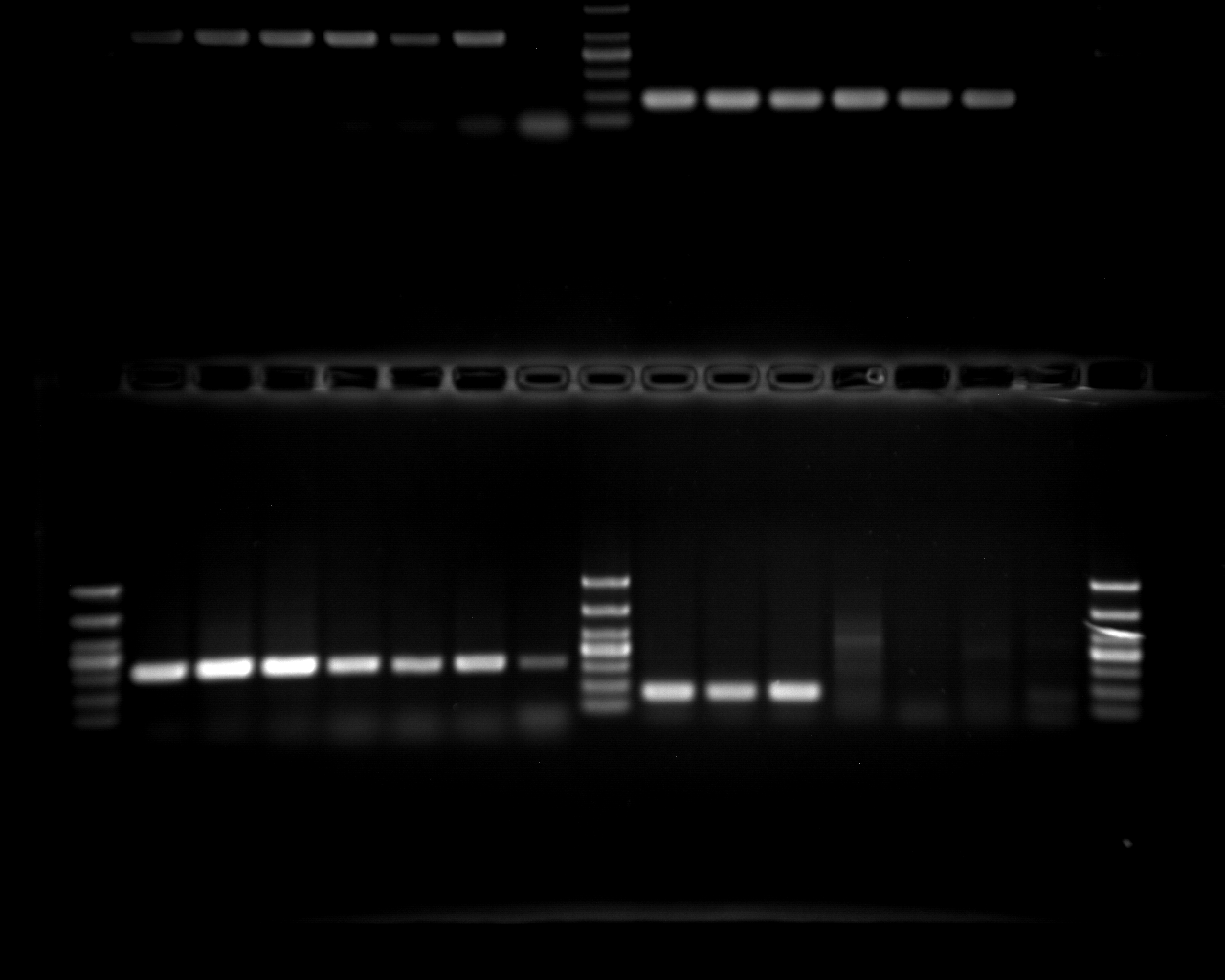

Supplement: Supplementary file 9 [file Presentation_1.ZIP › ╠⌠╤í/OBP4-second group.tif]

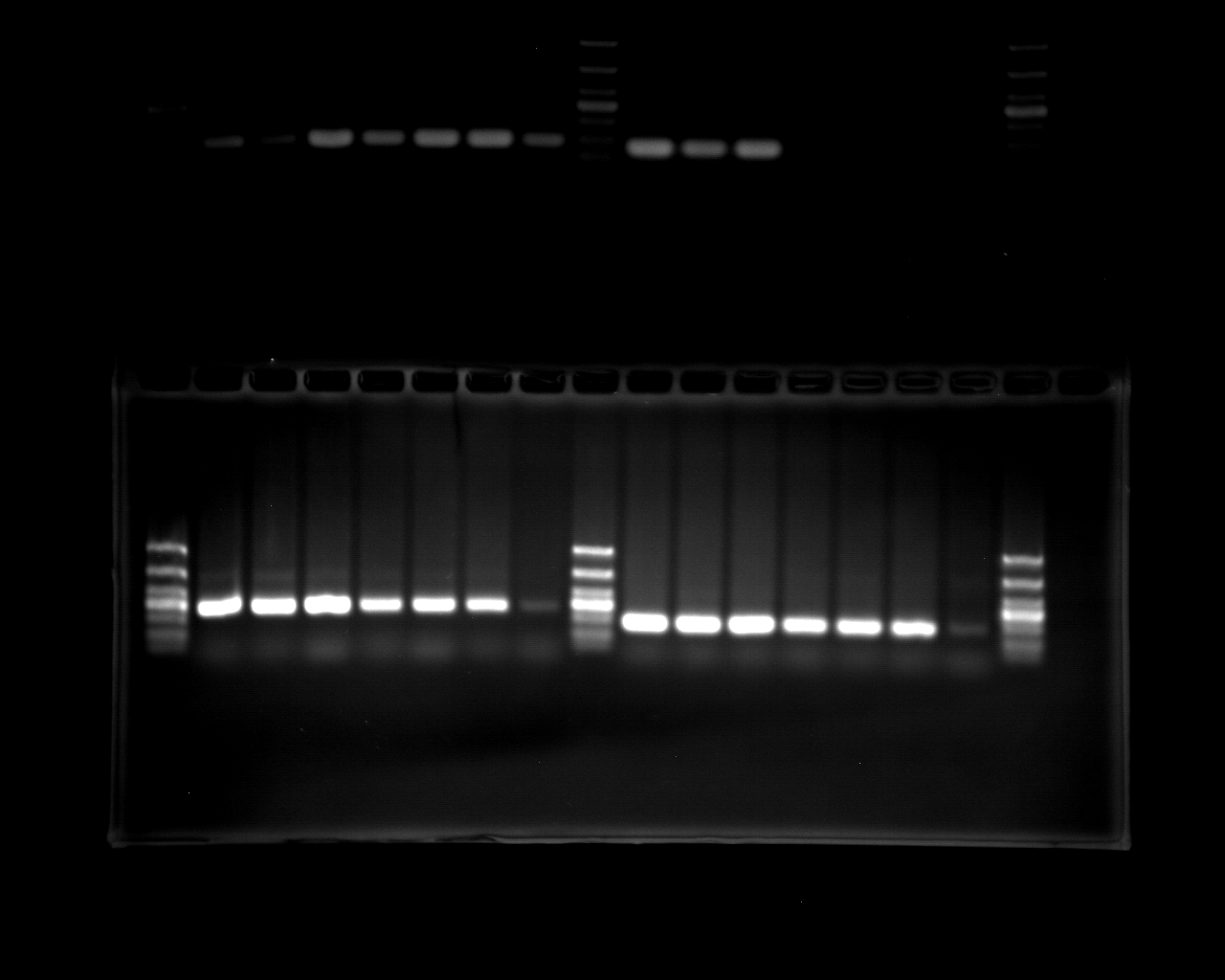

Supplement: Supplementary file 9 [file Presentation_1.ZIP › ╠⌠╤í/OBP5-first group.tif]

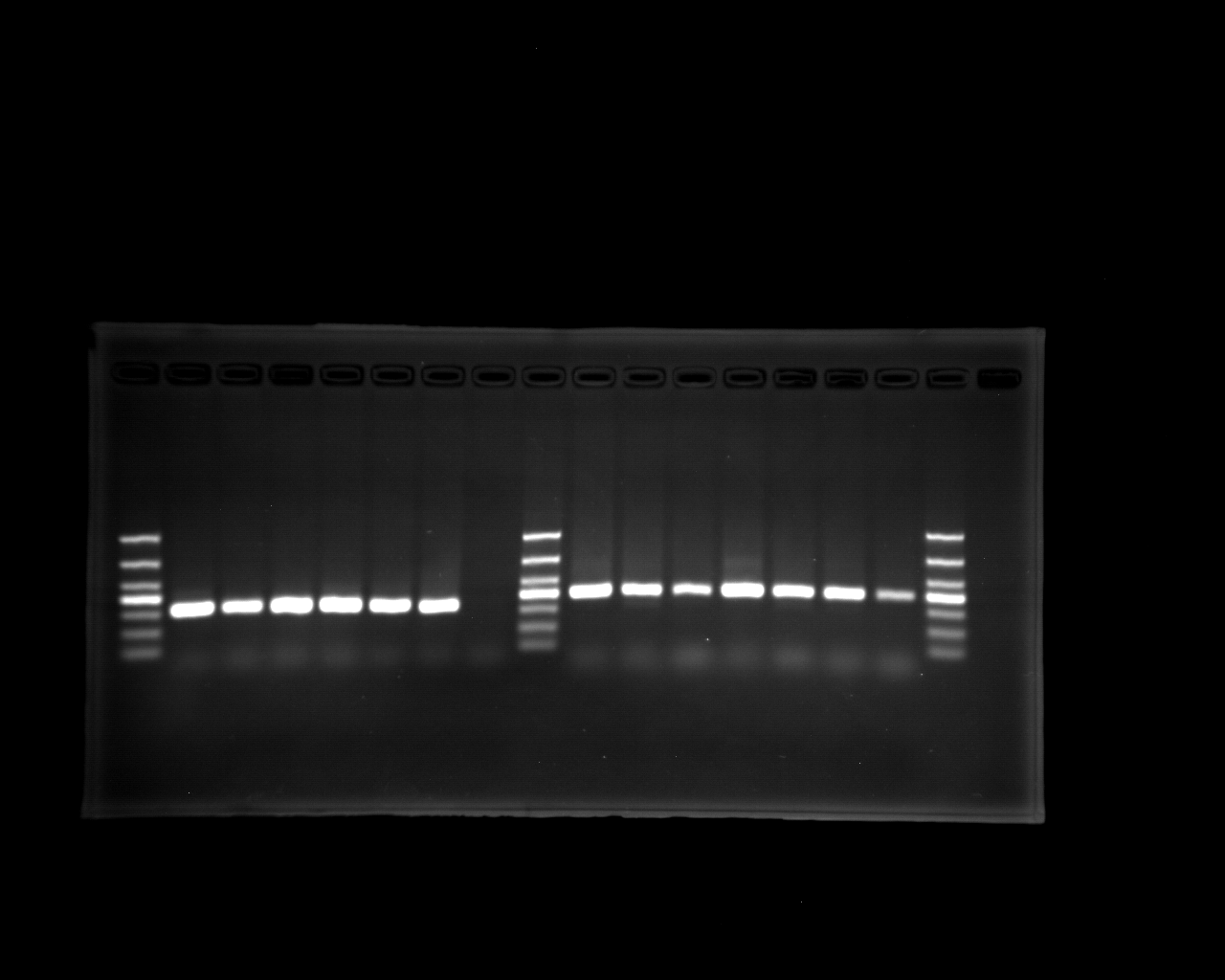

Supplement: Supplementary file 9 [file Presentation_1.ZIP › ╠⌠╤í/OBP6-second group.tif]

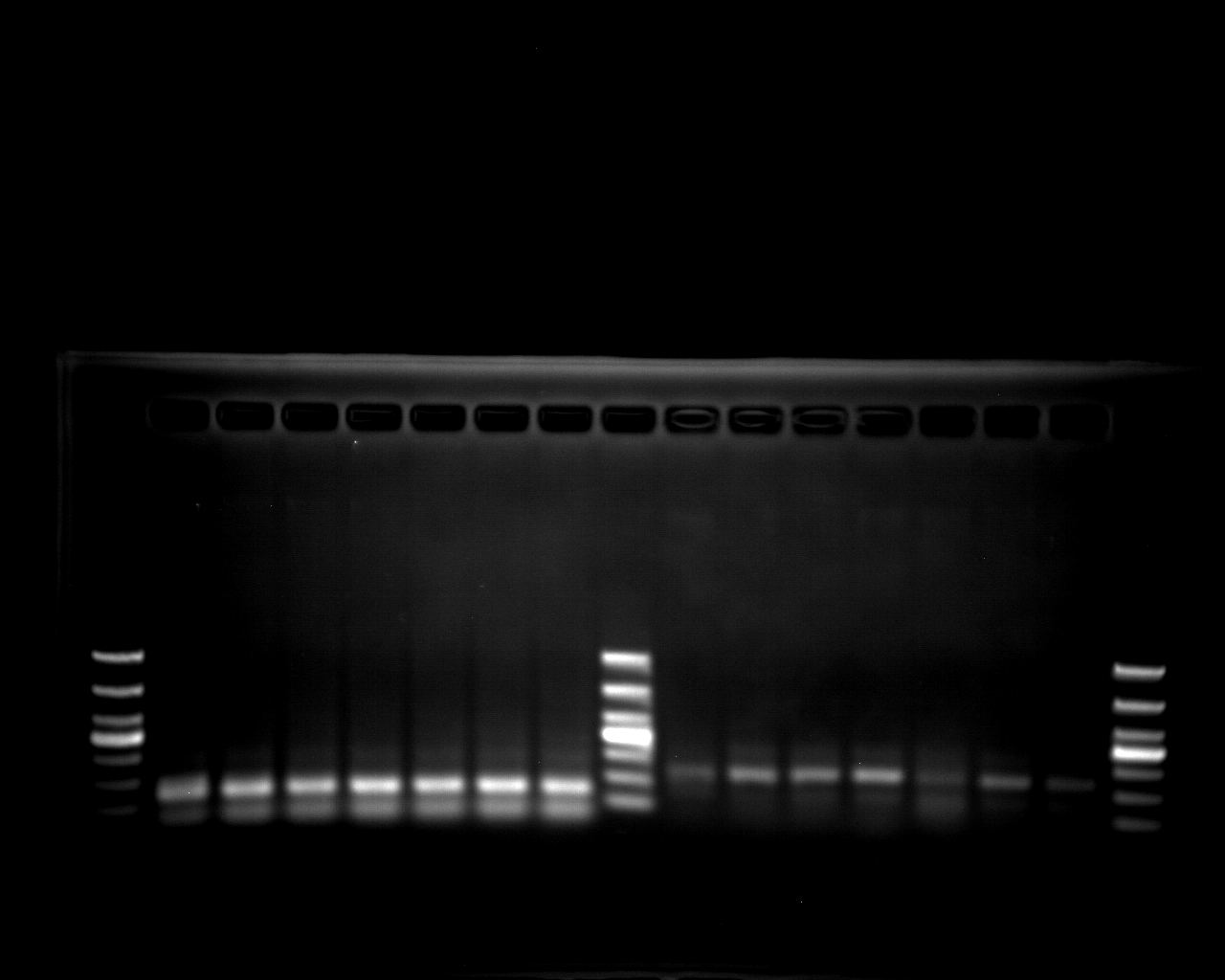

Supplement: Supplementary file 9 [file Presentation_1.ZIP › ╠⌠╤í/OBP7-first group.tif]

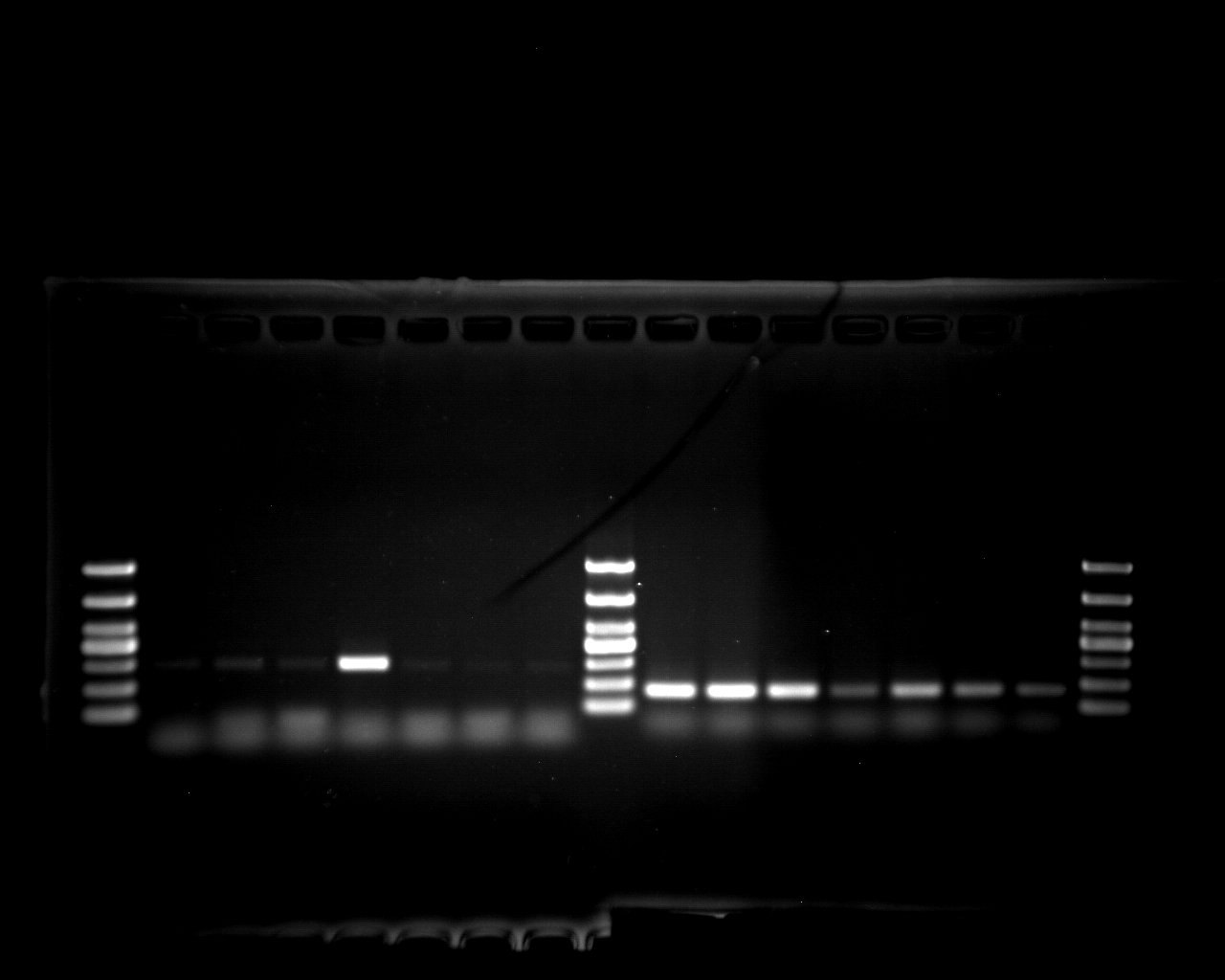

Supplement: Supplementary file 9 [file Presentation_1.ZIP › ╠⌠╤í/OBP8-first group.tif]

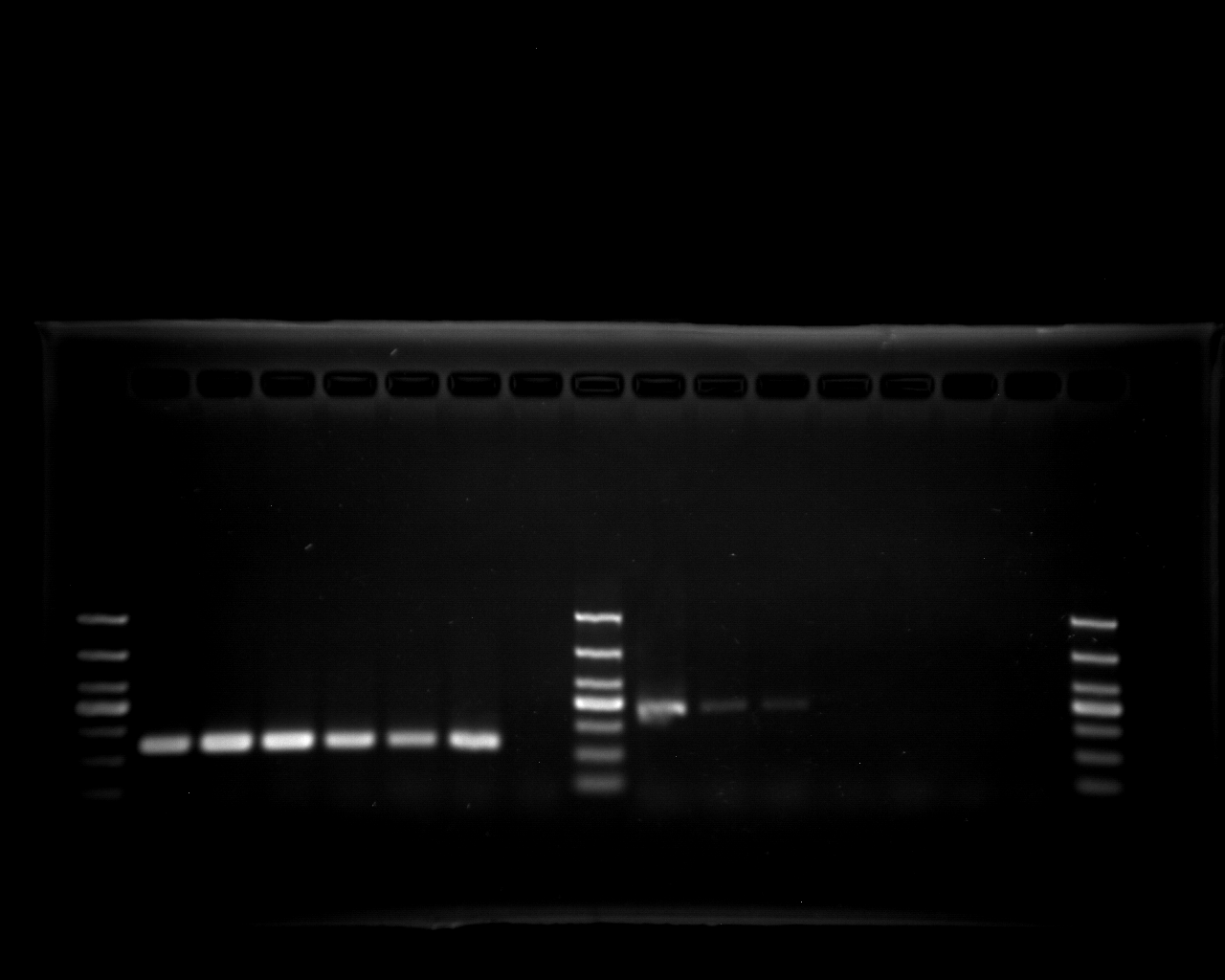

Supplement: Supplementary file 9 [file Presentation_1.ZIP › ╠⌠╤í/OBP9-first group.tif]
