# Supplementary material for: Expressions of Olfactory Proteins in Locust Olfactory Organs and a Palp Odorant Receptor Involved in Plant Aldehydes Detection
Source: Front Physiol. 2018 Jun 4;9:663. doi: 10.3389/fphys.2018.00663 (PMC5994405; doi:10.3389/fphys.2018.00663)
Supplement: TABLE S1 — Details of odorants used in EAG and EPG tests. [file Table_1.DOCX]

Table S1 Details of odorants used in EAG and EPG tests

|  | **Odorants** | **CAS No.** | **Solvent** | **Purity** |
| --- | --- | --- | --- | --- |
| 1 | E-2-pentenal | 1576-87-0 | paraffin oil | 99% |
| 2 | 2-Phenylethyl alcohol | 60-12-8 | paraffin oil | 99% |
| 3 | 5-Hexenenitrile | 5048-19-1 | paraffin oil | 99% |
| 4 | Pyridine | 110-86-1 | paraffin oil | 99.9% |
| 5 | E-2-methyl-2-butenal | 497-03-0 | paraffin oil | 96% |
| 6 | 1-Penten-3-ol | 616-25-1 | paraffin oil | 99% |
| 7 | Hexanoic acid | 142-62-1 | paraffin oil | 99.5% |
| 8 | Diacetone alcohol | 123-42-2 | paraffin oil | 99% |
| 9 | β-Ionone | 79-77-6 | paraffin oil | 97% |
| 10 | Octanoic acid | 124-07-2 | paraffin oil | 98% |
| 11 | 4-Allylanisole | 140-67-0 | paraffin oil | 98% |
| 12 | 2-Ethylfuran | 3208-16-0 | paraffin oil | 99% |
| 13 | 2-Pentanone | 107-87-9 | paraffin oil | 99.5% |
| 14 | Myrtenol | 515-00-4 | paraffin oil | 95% |
| 15 | Verbenone | 1196-01-6 | paraffin oil | 94% |
| 16 | Methyl Jasmonate | 39924-52-2 | paraffin oil | 95% |
| 17 | Safranal | 116-26-7 | paraffin oil | 90% |
| 18 | Crotonitrile | 4786-20-3 | paraffin oil | 99% |
| 19 | Hexanal | 66-25-1 | paraffin oil | 99% |
| 20 | 1-Hexanol | 111-27-3 | paraffin oil | 99% |
| 21 | E,E-2,4-hexadienal | 142-83-6 | paraffin oil | 95% |
| 22 | 1-Octen-3-ol | 3391-86-4 | paraffin oil | 98% |
| 23 | 2-Ethyl hexanol | 104-76-7 | paraffin oil | 99.6% |
| 24 | Methyl salicylate | 119-36-8 | paraffin oil | 99% |
| 25 | E,E-2,4-heptadienal | 4313-03-5 | paraffin oil | 90% |
| 26 | (+)-α-Pinene | 7785-70-8 | paraffin oil | 99% |
| 27 | β-Caryophyllene | 87-44-5 | paraffin oil | 98.5% |
| 28 | E,Z-2,6-nonadienal | 557-48-2 | paraffin oil | 95% |
| 29 | (−)-β-Pinene | 18172-67-3 | paraffin oil | 99% |
| 30 | 2-Nonanone | 821-55-6 | paraffin oil | 99% |
| 31 | Z-3-nonen-1-ol | 10340-23-5 | paraffin oil | 95% |
| 32 | 1-Heptanol | 111-70-6 | paraffin oil | 99.5% |
| 33 | 2-Octanone | 111-13-7 | paraffin oil | 98% |
| 34 | 4-Ethylbenzaldehyde | 4748-78-1 | paraffin oil | 98% |
| 35 | Pentyl acetate | 628-63-7 | paraffin oil | 99% |
| 36 | 2-Octanol | 123-96-6 | paraffin oil | 97% |
| 37 | Butyl acrylate | 141-32-2 | paraffin oil | 99% |
| 38 | Heptaldehyde | 111-71-7 | paraffin oil | 95% |
| 39 | Methyl phenylacetate | 101-41-7 | paraffin oil | 98% |
| 40 | 3,4-DMB  (3,4-Dimethylbenzaldehyde) | 5973-71-7 | paraffin oil | 98% |
| 41 | Nonane | 111-84-2 | paraffin oil | 99% |
| 42 | Hexyl butanoate | 2639-63-6 | paraffin oil | 98% |
| 43 | 2-heptanone | 110-43-0 | paraffin oil | 98% |
| 44 | E-2-hexenal | 6728-26-3 | paraffin oil | 99% |
| 45 | Butyl butyrate | 109-21-7 | paraffin oil | 98% |
| 46 | 3-Carene | 13466-78-9 | paraffin oil | 90% |
| 47 | 3-Hexenyl hexanoate | 31501-11-8 | paraffin oil | 98% |

All the chemicals' concentration used in this study is 1% (vol/vol).
