# Supplementary material for: Expressions of Olfactory Proteins in Locust Olfactory Organs and a Palp Odorant Receptor Involved in Plant Aldehydes Detection
Source: Front Physiol. 2018 Jun 4;9:663. doi: 10.3389/fphys.2018.00663 (PMC5994405; doi:10.3389/fphys.2018.00663)
Supplement: TABLE S2 — Details of OBPs in Locusta migratoria and the primers used for qPCR. [file Table_2.DOCX]

| **Name of OBPs in our work** | **Accession number** | **Forward primer**  **(5’-3’)** | **Reverse primer**  **(3’-5’)** |
| --- | --- | --- | --- |
| LmigOBP1 | ACR39387.1 | GGACCACACATGCCGCTC | GGATCTGGTAAGCTGTTTCGC |
| LmigOBP2 | ACR39388.1 | CACCGAAGATGAGCTGAAGGG | TGCCATCCTTTACCACTCCAA |
| LmigOBP3 | ACR39392.1 | GTCTTCAGATGGGCAGTACGA | TTCTCGTCGGGTTTGTCCTTG |
| LmigOBP4 | AEV45802.1 | ATGGCACAACACCGACATCC | GATCCCGTCTCATCCAGCAA |
| LmigOBP5 | AFL03411.1 | CACTTAATGCCTGCAAGGGTG | GTAGTCCTTTGGTGCCTGCTT |
| LmigOBP6 | AEX33162.1 | TCAGGGAATACGCCAAGACTG | CGTCAGAGAACAGCCGCATAG |
| LmigOBP7 | AEX33163.1 | ATGGAGGGCATAAAGGCGTG | GCCGACGAAACACTTCTCCT |
| LmigOBP8 | AEX33164.1 | GGTGATACAACACTGCAACGA | GGCAGAACACGAAACACTTGA |
| LmigOBP9 | AEX33165.1 | AAGAACCAGGCGGACAAGATG | TTCGCAGTCATCATCAACTCCA |
| LmigOBP10 | AEX33166.1 | TCAATGTCTCGGGCTGAAGAA | TCCGTAGTCGTTCTCGTTCTG |
| LmigOBP11 | AEX33167.1 | GAACCAACAAGGAGGCGAGA | CAGGACACCCGTTGATGTGA |
| LmigOBP12 | AEX33168.1 | GCTGTAACTCTGCTGGTAAGC | TTAGCATTAAGACCCGCGATGA |
| LmigOBP13 | AEX33160.1 | CGGTCCTACAACGAGGCAAA | TGTTGGGGTGGGCTTTCATC |
| LmigOBP14 | AEX33161.1 | GTCAACGAATGCAACGACACA | CGCCACCAGATCATTTTCGTC |
| LmigOBP15 | KU865299.1 | CACGATACCAGACGAGAACGA | GTCCAGTTGAGACTTCCGTCC |
| LmigOBP16 | KU865300.1 | TGGAACAGTGGTGGAATGGG | CATCTACCGCTCCCACGAA |

**Table S2.** Details of OBPs in *Locusta migratoria* and the primers used for qPCR

Note: LmigOBP13 and LmigOBP14 in our work are LmigOBP4 and LmigOBP5 in Genbank registered by Wang et al., 2015.
