# Supplementary material for: Expressions of Olfactory Proteins in Locust Olfactory Organs and a Palp Odorant Receptor Involved in Plant Aldehydes Detection
Source: Front Physiol. 2018 Jun 4;9:663. doi: 10.3389/fphys.2018.00663 (PMC5994405; doi:10.3389/fphys.2018.00663)
Supplement: TABLE S3 — Details of ORs in L. migratoria and the primers used for qPCR. [file Table_3.DOCX]

**Table S3.** Details of ORs in *Locusta migratoria* and the primers used for qPCR

| **Name of ORs in our work** | **Accession number** | **Forward primer**  **(5’-3’)** | **Reverse primer**  **(3’-5’)** | **Name of ORs in Wang’s work 2015** |
| --- | --- | --- | --- | --- |
| **LmigOR142** | **KU865301** | **TCACCCTGTTGCTCGTATGG** | **AGTCGTGTTCGTAATCGGCA** |  |
| LmigOR12 | KP843312.1 | GCGACACTACAGCCCGAAT | GGTTTCGTGCCCTTTCGTG | LmigOR6 |
| LmigOR13 | KP843241.1 | TCAGAGTGGGGAGCTGACTG | ACCGTGATCTTGAGAGGCTT | LmigOR133 |
| LmigOR14 | KP843367.1 | TCGACGCGTTCCACAAGAAG | GAACCAGACGAAGGAGAGCA | LmigOR50 |
| LmigOR15 | KP843258.1 | GATACCGCTGACGACTGCTC | TGGCGATGACTTGGAAACAG | LmigOR95 |
| LmigOR16 | KP843328.1 | CGTGTCTCTCCACCAGCGCA | GACGAAGAACTTGCCGGCGC | LmigOR10 |
| LmigOR17 | KP843365.1 | GTGGAGTTCCGCTCAGTTCTT | GCCACTGTCATAAACTGCCTC | LmigOR5 |
| LmigOR18 | KP843196.1 | GGAGTCGGTCCTCAAGTGG | TGACGATGAAGACGAACGAGG | LmigOR40 |
| LmigOR19 | KP843237.1 | GGGAGTTGACGCGAGATTAG | TGCCGTTGAACAAGTGTCGT | LmigOR39 |
| LmigOR20  LmigOR21  LmigOR22 | KP843270.1  KP843321.1  KP843252.1 | GGAGAGACTGGCAACGGAA AGCTGTACCCCATCAACAGGA  GGCAGACCTTCCTGCACTT | TCGAATGGCCTCCACGAGT ATCTCTGGTCTGGCGGAGTAA  TTGCTGTTCAGGTGGTGGAG | LmigOR105  LmigOR43  LmigOR85 |

**Note:** *LmigOR142* is reported for the first time in this work.
