# Supplementary material for: Expressions of Olfactory Proteins in Locust Olfactory Organs and a Palp Odorant Receptor Involved in Plant Aldehydes Detection
Source: Front Physiol. 2018 Jun 4;9:663. doi: 10.3389/fphys.2018.00663 (PMC5994405; doi:10.3389/fphys.2018.00663)
Supplement: TABLE S5 — List of OBPs of L. migratoria and 6 other orders of insects used for the phylogenetic tree. [file Table_5.DOCX]

**Table S5: List of OBPs of *L. migratoria* and 6 other orders of insects used for the phylogenetic tree.**

>LmigOBP1

WDVNMRLTGRIMDAAKEVDHTCRSSTGVPRDMLHRYAEGQTVDDDDFKCYLKCIMVEFNSLSDDGVFVLEEELENVPPEIKEEGHRVVHSCKHINHDEACETAYQIHQCYKQSDPELYSLVVRAFDATIGD

>LmigOBP2

MEMTPEFMEIVNKCKTEHEPTEDELKGMMALKVPESANGKCFMGCVLQEIGVVKDGKFDKEEAKKHAASKMTDKDELEKHMQLIEKCSQEVGGETDSRGIGPKLMECIKQFAPEFDIALPQQPSE

>LmigOBP3

DAEKMKEAVDKCKASENLDSLDGLKSSKSPSTEEEKCFIGCLMMDMKLLSSDGQYDAASTKDMINNCEYLKDKPDEKSAALEVADDCAGKATGCSGHCECGPKAVGCLIKGMVDKGYEESFARIDKMLEKLDD

>LmigOBP4

DDVWHNTDIPATMAECNATFRLGWRCWDNLLSDGHVIDESKYQQKCWFYCLLDETGSMHADGAFDKDLLKTVLQGFPNGSSLAHLDETTYTCVAQRNEVDLCERAYAVVKCIMTEELSRMHQSS

>LmigOBP5

DDEMREMMDQLHQTCVGESGVSEGNIDAARKGNFIDDGNLKCYMKCIFVQMTCMSDDGVFDADTAIAMLPDNLKDVASKALNACKGEKGSDACDTAFKINQCLFKQAPKDYILV

>LmigOBP6

EKQAPWCPTTASQGVQEDMGQCAEEIKDAILREYAKTVSNRRTRSAEMSEEDRLLVGCMVSCLFRKGPHSRLQTGSKLLLAELGAMRLFSDGADDARYRNATATAVRRCSASSRSLLPDDGGPRHECELGFFMFECVSDQITEYCQWQPE

>LmigOBP7

DEAAETKVMEGIKACMASEHLGSLGQLKANNEARTPEEKCFVGCVMKHLHVLNSEGQYDLALVKERASSCPELDKDPQKKADTLRVAEECATKVIGCSGYCECGVAAGECLAQGMEAKGHETIYDFLRKIVDKMDV

>LmigOBP8

PSITSTEMRMDMMVIQHCNETHPVALIDMNKALINKKIEPQNTVFKCFVFCLLNKYEWMDDEGGFLIANMKHNLSDSHLDQLSIDFIVYKCSATGSSDKCERAYRFTECFWGEVTKFPENSDEKYEDPDLFALYQ

>LmigOBP9

LSLEQLRQTSKIVRNMCLKKTGVDLALVEGIQEGQFPDNQDLKCYMKCCMGAMQVLRQGRYNVNAAKNQADKMLPPDLKGRFIDMLDACSDRGDGVDDDCEMAYQLTKCSYETDKEIFLFP

>LmigOBP10

AISESMSRAEEAASKIDLPELFEECNETFTIPKVTLNYFFSHGRLQNENDYGSKCFIHCLTDRSGEIDSDGNFDVDLIKVMTRRFPNETNIEGLNEMVETCVADRGETDFCERAYGLVSCLVKEKLARLGNSH

>LmigOBP11

EPDFTKGISDVKACMASENLDSLDALRTN KEARTAEEKCFIGCIMKFVEVLNSDGQYDVALFKDHINGCPEMAKDQQKKAALLEVAESCAGKASACSGHCECGVIVANCLADEMEAKGQETIYDLLETIFAKMDA

>LmigOBP12

AMVTTEIPTEDILQRVQVCNKTYPVSQEMLRSLASTGGLLSDESDVNTRCYLECYERLGGTVNKDGKFNPEKAVTLLVSYYPKIAELGVDSVTEILKNCNSKSGTGQCMTSYLIRNCFIAGLNAKSPHTSVFDTSSSHI

>LmigOBP13

EDSLMEIVIREVKGCMDSEHLNSIADLRSYNEAKSPEEKCFLGCMLKKFKALDADGQYDAEGLKATIQHCPRMKAHPNIQQAALQVADECAGKVTGCSDYCTCAPLATRCLHEGMKNKSFQTIFIALDEALDKMQS

>LmigOBP14

GEVFTMSQLKAAVNECNDTYFLSQKNWDTVFTTGSLEDENDLVAKCFFECVLEKTGAMDEKGNINSDITKAVFLASHEGTGTAVQGHDDLIDMCVPGRDETDICERGYALVKCVTVEELSRRQARK

>LmigOBP15

VNVDIETIWRECNETFPASEEALISFGKNGTIPDENDSVARCFTDCYGKKTTLLTSDGSLNWTTLDFLMRSYDMKPTAKETFGKCQKNTSNVECMKSYLSLRCVAETVESLTDIR

>LmigOBP16

LKCHTDEDTQNPDEFQEVAAMCMKNTSGSELNRNDRESKRNGNNYHKTNFENTNDNWNSGGMGQTFPGYNSENEGYGFRGSGRCNANGDGYNGNRNNMNQNNMNGMRQRPRNRNRRSGQQSETADVDLEDIEPCAVHCIFRQMGMLGDDAIPDRSAVAKVMLRGVKDTEVKDFVQEAVEDCFDQVESDRKGSKCEFSKNVALCLRQKGRENCEDWGEQDGDQQSNQNKNGNNNGNYSNNSNQYGNKKWN

>AlinOBP1

DEQTNAMVAKAFNKCREEFPISDDEIGGVREKTTIPESHNAKCLMACMLREGKMLRDGKYEKENALIMADVLNKDDPASADKAKQLVETCAGKVGTDAGGDECEFAYKMAVCAAEEAKKLGVRPPDF

>AlinOBP2

YQEQLKQTIRDCQDGKEVTDDELEEFTKPLIPRNREEKCIMACVMRTYNIISNGHYDPKIAFGILKGILKDHPEKLNKIKEVMDHCGEDVPSHMDDECDLAGEIMQCEVKYQKAMGMA

>AlinOBP3

ISKEYSARMIAAKEKCQKEFNVTDSVVEDFMKRNIKPESKSGKCMVHCIMEEMGMIDDHKINTEQVKLGNKEKWDDPALVELANQVADTCDQEVFTEGRCKCLVAVEYMMCLATHGDEVGLPHVDFEDSQDS

>AlinOBP4

GELPEEMKEMAQGLHDSCVEETGVDNGLIAPCAKGNFADDAKLRCYFKCVFGNLGVISDEGELDAEAFGSILPDSMQELLPTIKSCGGTTGSDPCDLAMNFNKCLQKADPVNFLVI

>AlinOBP5

AMSQAQMKQAMKTVRNMCIPKSGVDKEALAKMVNGEFDESDQKLKCYLGCVLGMMQAVKNNKINLTMVRNQITKMLAPERGQRILAAFESCATVTGDDNCGLAFRFAKCIYDTDKEAFIVP

>AlinOBP6

KELTDEQKEQIFAEIKNCMESTKLTDEEFESIMAKKELPTSIEGKCFTKCLMEKMEYLEEGGKINVIAVQAGMEENMEKESEITKAKEVIQQCADSVPPEDSCEYAYGISQCMYNKMKEAGISGS

>AlinOBP7

QQEDCKTAPAGWPRRPPQCCDLPFPLEGMKKEFGSCIRQIGNRQSSAVPTAQAVRDARLCIEECVYKGLGFMDEHKLNKDQLLEQLKKGIADKKDWTKPMEGAVKRCHETITKRETPQEAACQDSAHEFTHCAMRELFLNCPASEWNNNDECNLVKSRMQACPNIPPPPPPPPQGFRGQGPPPQ

>AlinOBP8

VINKDYLEKVVTAKDKCLKEFNVDDSVVEDFIVKYNKPQSESGKCMVACFMEERGMMKDGKTITEQVMLDNQEKWIAATHVNMGKEVIDTCDKEVPNEENDKCDLAVDYMMCLVKRGDEAGLPKMDVAQLKH

>AlinOBP9

SQRTKQQPKSKTKESVVGATRPRDAKATECVNKVNANEEESASFFRKEIPETEAGKCLLACYLEGKGLIVGGKISSSGAARVAARAYPNNRVKTGNVKHILSHCGTIAGRESNNCEMAYKLADCTTTLSDKFRL

>AlinOBP10

QELPPPGDVKNKTVVFKNSFLRSAKYCSSIYETSTLAIMALLMSEKSDDQNGKCFLNCMLQRYRLMSQDGSYNKDKFKPFLEYIPDSKFLQSIRGNLKNCISEKDPDPCEKASKFIKCFYTRARNKGEIGASKEVIPADGF

>AlinOBP11

ISKEYHDKAIEAKNTCAKLHNVDDETIMTYWKNHQLPEKEPETCIVICYLKEMKLVVDGKVDADAWKASNKEKWDDEKHVAAADEIVDKCSAEVPPTENECEWGLALTKCALKHGKEAGIPPPDMEHPKRR

>AlinOBP12

YQELLKETIKKCQNGRDVTDDEVEEFTKPLVPKNEEERCLVACVFKEYKVIIDGHFDPVNALNVAKVVYKDYPDKVERIKDVLDHCGEDIPTHNDNECDLAGDIMKCEVKYLNSVPKMTSLEFLAGSMAATAEP

>AlinOBP13

LDGILPQANQDECREESNFRGELNDDVGRNVTQELKCFAACSLMKLGIMNEKDGTVNMTRLDELIASHTPGKDAADVFKTTVVEPCMKEVKKSTDYCEYSYQLIACGMSKVP

>AlinOBP14

RKIAHECADECLYKSSNLLTSAGELDKDAIKALVTKLYTGDWATAATTAIDKCLASAKGEVEATSKCKSGSFQLSRCFMRSMFLGCPASSWTESTECAAAKARLTKCPNAMAPMPHKK

>AmelASP1

APDWVPPEVFDLVAEDKARCMSEHGTTQAQIDDVDKGNLVNEPSITCYMYCLLEAFSLVDDEANVDEDIMLGLLPDQLQERAQSVMGKCLPTSGSDNCNKIYNLAKCVQESAPDVWFVI

>AmelASP2

IDQDTVVAKYMEYLMPDIMPCADELHISEDIATNIQAAKNGADMSQLGCLKACVMKRIEMLKGTELYVEPVYKMIEVVHAGNADDIQLVKGIANECIENAKGETDECNIGNKYTDCYIEKLFS

>AmelOBP3

DDITLCLKQENLNLDDIDSLLEDESERMLRKRGCIEACLFHRLALMNDNVFDVSKFDVYLNDTDMDMDLKDSIRKIIRQCVDNAKNEDKCLTAQKFSRCVIDYVKFHITQYMISNANSNTTSEEESSDNST

>AmelASP4

DTVAILCSQKAGFDLSDLKSMYESNSEEQMKKLGCFEACVFQKLHFMDGNTLNVEKLESGTRELTPDDFTEDVHEIIEQCVSKAADEDECMVARKYIDCALEKMKFLDDELEKIAGN

>AmelASP5

MSADQVEKLAKNMRKSCLQKIAITEELVDGMRRGEFPDDHDLQCYTTCIMKLLRTFKNGNFDFDMIVKQLEITMPPEEVVIGKEIVAVCRNEEYTGDDCQKTYQYVQCHYKQNPEKFFFP

>AmelASP6

KKMTIEEAKKTIKNLRKVCSKKNDTPKELLDGQFRGEFPQDERLMCYMKCIMIATKAMKNDVILWDFFVKNARMILLEEYIPRVESVVETCKKEVTSTEGCEVAWQFGKCIYENDKELYLAP

>AmelOBP7

NGINEILKIMAVSMKDIRYCIIHMGLTFKDFIKMQELLQEEDISEGNIKKYLTNYSCFITCALEKSHIIQNDEIQLDKLVEMANRKNISIDVKMLSECINANKSTDKCENGLNFIICFSKLLSDMYEDTFEDTLKHKSYV

>AmelOBP8

MTIEELKKTIKNLRKVCSKKNDTPKELLDGQFRGEFPQDERLMCYMKCIMIATKAMKNDVILWDFFVKNARMILLEEYIPRVESVVETCKKEVTSTEGCEVAWQFGKCIYENDKELYLAP

>AmelOBP9

DIKKDCRKESKVSWAALKKMKAGDMEQDDQNLKCYLKCFMTKHGILDKNAEVDVQKALRHLPRSMQDSTKKLFNKCKSIQNEDPCEKAYQLVKCYVEFHPEVLQTVPFL

>AmelOBP10

GTRPSFVSDEMIATAASVVNACQTQTGVATVDIEAVRNGQWPETRQLKCYMYCLWEQFGLVDDKRELSLNGMLTFFQRIPAYRAEVQKAISECKGIAKGDNCEYAYRFNKCYAELSPRTYYLF

>AmelOBP11

EISDIDEFREMTSKYRKKCIGETKTTIEDVEATEYGEFPEDEKLKCYFNCVLEKFNVMDKKNGKIRYNLLKKVIPEAFKEIGVEMIDSCSNVDSSDKCEKSFMFMKCMYEVNPIAFIAP

>AmelOBP12

RSVNIFQDIADCVDRSNMTFHELKKLRDSSEARIKLINEEENFRNYGCFLACIWQQTGVMNGSELSTYNIAGIIEGQYHDDEDLKTFFHKIALTCEDDVHRKFLHVNDECDVALSFKLCMLKAMRNYP

>AmelOBP13

VSEESINKLRKIESVCAEENGIDLKKADDVKKGIFDKNDEKLACYVDCMLKKVGFVNADTTFNEEKFRERTTKLDSEQVNRLVNNCKDITESNSCKKSSKLLQCFIDNNLMKIFE

>AmelOBP14

LTIEELKTRLHTEQSVCKTETGIDQQKANDVIEGNIDVEDKKVQLYCECILKNFNILDKNNVFKPQGIKAVMELLIDENSVKQLVSDCSTISEENPHLKASKLVQCVSKYKTMKSVDFL

>AmelOBP15

LSIKDFQNAIRMGQSICMAKTGINKQIINDVNDGKINIEDENVQLYIECAMKKFSFVDKDGNFNEHVSREIAKIFLNENEINQLITECSAISDTNVHLKITKIFQCITKFKTINDILNS

>AmelOBP16

MTHEELKTGIQTLQPICVGETGTSQKIIDEVYNGNVNVEDENVQSYVECMMKKFNVVDENGNFNEKNTRDIVQAVLDDNETDQLIVECSPISDANVHIKISKIFQCFMKYKTITDILNS

>AmelOBP17

MTLDELKSGLHTVQSVCMKEIGTAQQIIDDINEGKINMDDENVLLFIECTMKKFNVVDENANFNEKISSDIVRAVLNDNEADQLLAECSPISDPNALIKISKILECFFKYKTINQILNS

>AmelOBP18

LTLEEFQIGLRAVVPICRIETSIDQQKEDDFRDGNIDVEDEKVQLFSECLIKKFNGYDDGGNFNEVVIREIAEIFLDENGVNKLITECSAISDADLAVKSAKLLKCIGKYKTLKEMLSG

>AmelOBP19

MTIEELKIQLRDVQEICKAESGIDQQTVDDINEVNFDVEDEKPQRYNECILKQFNIVDESGNFKENIVQELTSIYLDENVIKKLVAECSVISDANIYIRFNKLVKCFGKYKTMKEVLNL

>AmelOBP20

MTIEELKIQLHDVQEICKTESGIDQQTVDDINEVNFDVEDEKPQRYNECILKQFNIVDESGNFKENIVQELTSIYLDENVIKKLVAECSVISDANIYIRFNKLVKCFGKYKTMKEVLNL

>AmelOBP21

LTLEELQIGLRAVIPVCRIDSGIDEKKEDDFRNGIIDVENEKVQLFSECLIKKFNAYDDGGNFNEVVVREIAEIYLDENEVNKLITECSAISDADIHLKSSKLIKCFAKYKTLKEIMNE

>BmorOBP1

DVYVMKDVTLGFGQALEQCREESQLTEEKMEEFFHFWNDDFKFEHRELGCAIQCMSRHFNLLTDSSRMHHENTDKFIKSFPNGEILSQKMIDMIHTCEKKFDSEPDHCWRILRVAECFKDACNKSGLAPSMELILAEFIMESEADK

>BmorOBP2

TAEVMSHVTAHFGKTLEECREESGLSVDILDEFKHFWSDDFDVVHRELGCAIICMSNKFSLMDDDVRMHHVNMDEYIKGFPNGQVLAEKMVKLIHNCEKQFDTETDDCTRVVKVAACFKKDSRKEGIAPEVAMIEAVIEKY

>BmorOBP3

SQEVMKNLSLNFGKALDECKKEMTLTDAINEDFYNFWKEGYEIKNRETGCAIMCLSTKLNMLDPEGNLHHGNAMEFAKKHGADETMAQQLIDIVHGCEKSTPANDDKCIWTLGVATCFKAEIHKLNWAPSMDVAVGEILAEV

>BmorOBP4

SRDVMTNLSIQFAKPLEACKKEMGLTETVLKDFYNFWIEDYEFTDRNTGCAILCMSKKLELMDGDYNLHHGKAHEFARKHGADETMAKQLVDLIHGCSQSVATMPDECERTLKVAKCFIAEIHKLKWAPDVELLMAEVLNEVSWKS

>BmorOBP5

ESGVDVVKNLSLSFARFFLECDEERHFQPEVRLKVMTFWYSESSTWDRDVGCAFLCIFKKMEIDNPQDPSYRTHLELLSFANSEDNKIANQMVEIFYACGENTETDPCLWALEQVKCYKNRINQLGLTPTF

>BmorOBP6

SSEAMRHIATGFIRVLDECKQELGLTDHILTDMYHFWKLDYSMMTRETGCAIICMSKKLDLIDGDGKLHHGNAQAYALKHGAATEVAAKLVEVIHGCEKLHESIDDQCSRVLEVAKCFRTGVHELHWAPKLDVIVGEVMTEI

>BmorOBP7

AVTEEELKIEFTKLVMKCTKDHPVDMSELMQLQQLIAPKKTESKCLLACAYKLNGVMTSQGLYNLEHAYKIAEMSKNGDEKRLENGKKVADICVKVNDVEVSDGEKGCERAALIFKCTLENAPKVFKFGSSEYNCQ

>BmorOBP8

SSLDDLKMVYKNVIKECVGDYPITAADLKLIKARQIPNDDIKCVFACAYKKTGMMTEEGMLSVEGIKDMSQKYLSDNPEQLRKSKEFAEACSSVNDQQVSDGTKGCERAALIFKCSTEKITNFGFEL

>BmorOBP9

VSYEQKIKIRDQLDRAGFECFKDHKITEDDIKNLRANKPATGENVPCFIACVMKKTGVMNDQGVIRKGPVLELAKKVLADDKDIKKLQDYIHSCSHVNSETVHDKGKGCEFAMQAYTCMSANASKFGFNI

>BmorOBP10

VSDEQKIKIREQIDKSGFECFKDHKITEDDIKNLRARKPATGENVPCFIACVMKKTGVMNDQGVIHTEPVLQLAKKVLTDDKDIKKLQDYIHSCSHVNSKTVHDKGQGCEFAIQTYTCMSANASKFGFDV

>BmorOBP11

LTEEQKAEITKSSLPLIAECSKEFSVNQGDIDAAKKLGDPSGLNSCFVGCFMKKAGIINASGLFDVAATIEKSKKYLTSEEDLKAFEKLTETCAPENDKPVSDSDKGCERAKLLLDCFVANKGSFSVFSL

>BmorOBP12

LTDEQKNKIQSKFIEIGAECIVEHPISIDDINSFKNKKFPSGVNAGCFVACIFNKIGLFDDKGNLSHNSALEKAKGIFNADEEVKNLEEFLNRCAKVNGEAVGDGVKGCERAKLAYNCLIENSLEFGFNIDF

>BmorOBP13

VTPEESKAFEAFAKPVIEQCQKDFGMDKESFAQKNLDEIDECLIACVVEKFGITNDEKIDGDALKALVTKFVGNEEERNKINKIVEECTEDANKSGDGTCNTSTILFLCLLKNGKDLWGF

>BmorOBP14

MSRQQLKNSGKMLKKQCMGKNDVTEEEIGDIEKGKFIEQKNVMCYIACIYQMTQIIKNNKISYEASIKQIDLMYPPELKESAKASAGRCKDVSKKYKDICEASYWTAKCMYEDNPKDFIFA

>BmorOBP15

FVNTMTKQQIKNSGKILKKACISKNDVTEDQISDIDKGKFIEDKNVMCYIACVYSMSQVVKNNKFVHDAMVKQVDMMFPTEMRDAVKASIANCRGVAKNYKDICEASFWTAKCMYEFDPANFVFA

>BmorOBP16

MTRAQVKKTMTIMKNQCMPKNGVTEDQVGKIEEGIFLENHNVMCYIACVYKTIQVVKNDRLDKDLISKQIDVLYPQEIRESTKKAVGDCINLQEKYDDWCEGIFRSTKCLYEKDPANFIFP

>BmorOBP17

MTRQQLKNSGKIMKKTCMPKNDVTEEEIGQIEQGKFLEQRNVMCYIACIYTVTQVVKNNKLSYDAVIKQVDVMFPAEMRPAVKAAAENCKDISKTFKDICEASYWTAKCMYDFDPKNFVFP

>BmorOBP18

MTMKQIKNTGKMMRKSCQPKNNVDDEKINPINDGVFIEENEVKCYIACIMKMANTMKNGKLNFEAAMKQADLLLPDEMKEPTKEAIVACRKVADSYKDVCDASFHVTKCIYNHNPSVFFFP

>BmorOBP19

SKDHPVTTEELRMHKHELPDSKNAKCLMKCVFRKCNWLDSKGMYDINAAYASSTKDFSDDKTKQENANKLFDTCKSVNEENVGDGEEGCDRSLLLAKCLTKAAPQVSIYYS

>BmorOBP20

AHGQLDDEIAELAAMVRENCADESSVDLNLVEKVNAGTDLATITDGKLKCYIKCTMETAGMMSDGVVDVEAVLSLLPDSLKTKNEASLKKCDTQKGSDDCDTAYLTQICWQAANKADYFLI

>BmorOBP21

GKDKPVLSEEIKEIIQTVHDECVGKTGVSEEDITNCESGIFKEDVKLKCYMFCLLEEAGLVNDDGTVDYEMFTSLIPEEYFDRATKMIFSCKELDTPDKDKCERAFEVHKCSYEKDPDFYFLF

>BmorOBP22

LYTQKVAVSFPKDKTTIVVEAMKSCIAKTGANPNVIEVISSGKVSEDEKFKEFFYCACNDIGVVNPDGHIKVKECIELFPKETQPLVEPVIKNCDKEGVNKYDTLFKYLKCFQETSPVRVTLA

>BmorOBP23

EDSRKLVSFAPEVAKKLKVLIQECLNENGLGEDAIEVIRAGEYREDEPFQNLVYCAYKKFGALDENNRIISQVAAASFPKDIDVVTVIESCGKEDGNTPVEQVFKYFKCFQKNSPVRMQLY

>BmorOBP25

DNVHLNEDEREKANWYTAECGVETGVSTEVINAAKIGKYSKDKAFKKFVLCFFKKSAILNSDGTLNMVVALAKLPSGVNKSEAQSVLEQCKNKTGQDAADKAFAILQCFHKGTKTHILF

>BmorOBP26

DNVHLAETQKEKAKQYTSECVRESGVSTEAINAAKIGKYSKDKAFKNFVLCFFNKSAIFNSDGTLNMDVALAKLPPGVNKSEAQSVLKQCKNKTGQGAADKAFEIFRCYYKGTKTHILF

>BmorOBP27

DNVHLTETQKEKAKQYTSECVKESGVSTEVINAAKTGQYSEDKAFKKFVLCFFNKSAILNSDGTLNMDVALAKLPPGVNKSEAQSVLEQCKDKTGQDAADKAFEIFQCYYKGTKTHILF

>BmorOBP28

NGCVAVPFPKDKTIIIVEAMKSCIAKTGANPNFIDVIRSGKVSEDEKFKEFYYCTCNDTGFVNPDGHIKVKECIELFPKETQPLVEPVIKNCDKEEGVNKYDTLFKFLKCFQETSPVRVALA

>BmorOBP29

TTGCKNCVILGKEERAMFRSHSDACLAQSRVEPRLLESMMNGELIDDAALRKHVYCVLLSCKMIGKDGKLLKAAILGKLAARPAGRDVTKVLEACAEQPGASPEDVAWNIFRCGYNRKAVLFDYMPAGGASSGNTENHP

>BmorOBP30

EDYYYDIVTRDPDDLMREKENEVRALRAFQADCAEDVQVKPDLVVNLKSGDWQTEDVSLKKWALCVLMKLGLMTAQGVFKMNEAMSKIPDMNDKIIAEKLIDDCLSLQATTPHDAAWNYIKCHHQKDPEGNFSSLNIF

>BmorOBP31

LTDEQKENLKKHRADCLSETKADEQLVNKLKTGDFKTENEPLKKYALCMLIKSQLMTKDGKFKKDVALAKVPNAEDKLKVEKLIDACLANKGNSPHQTAWNYVKCYHEKDPKHALFL

>BmorOBP32

YNTKLFSHSLGSEPSLSILYARDKKSDKVTNECLMEMYPKNLYKYPLRIDRNDIPCIIHCVLKKFGIISNDGFINIKNYYRRVQAIHRYDPRILISDVGETCAQNINGMNLDHDVCKKAKVFNDCTQLYAISYREPEDW

>BmorOBP33

MYAHDKLSDMIADQCLNEMYPRSKRLEIEESDEPCIIFCVLKKFGIMSPTGVINLEAYRKRVQLPEQLAQRNSINDFGSACLESAEATQHKQDVCKKAKVFNECTHLYKILLK

>BmorOBP34

SRTRGSSGTLVDFTDPKVQGHLDALVRMAQSCVIKVRATPKDVRAYFTNSSPVSRSGQCFATCMLEQSDIINHGKVNRDLLVHLAGLVNGKNSRVVRKLNSVSRLCLDSISGMTDRCQLASTYNDCLNENMIEFAFPLDIAEEAVRKMPFHLIQPK

>BmorOBP35

GMSTHVLDFKRNMTECLKEVQNNDKRPIKRLSPKQESPIHGECLIACVLKKNGVIQNGKVNKDNLMALVSKFHAKETKLMKKLEKNLDRCINISVKNHDECSLASQLNDCTNDIMASSKQKILFNY

>BmorOBP36

FKPLTKDEHIERYNKMNEDIEPFRKNLTECARQVKASMADVEKFLKRIPQSNMEGKCFVACILKRNSLIKNNKLSQENLLEVNRAVYGDDSEVMSRLKTAILECSKIVEDIFEICEYASVFNDCMHMKMEHILDKITMERRMEALGQMSSNPDEWSEEEDEMLKLVKDEL

>BmorOBP37

EPEKENHFTLALKKTLFSTARSCMSHVNANETDLEYLRKDPPFPDKAACIIKCLLEKIGVVKNNKYSKMGFLTAVSPLVFTNKKKLDHYKSVSENCEKEINHDQTTECELGNEVVSCIFKYAPELHFKT

>BmorOBP38

RLKSTEAPKSKTALFNDQDNMGYEELDMEEIMSACNESFRIEYAYLESLNDSGSFPDETDKTPKCYIRCVLEKTEILSENGVLNPATAALVFAGERNGKPMSDLEEMAVACADRHEKCKCEKAYNFVKCLMYMEIDKYEKKN

>BmorOBP39

AISTDNEQRCKNPPTAPQKIERVITLCQDEIKLSILREALDVIKEEHTMPAERKRNKREVPFTHDEKRIAGCLLQCVYRKVKAVDGFGFPTLEGLVGLYSDGVNERGYFMAVLEASRECLMKNHDKFSRTTPMDNGRNCDVSFDIFECISDRIGEYCGTSGL

>BmorOBP40

MSEFIQPSWRTQCNFRLNWDNRNRLSIDISHGAATTQTPVPTTKPKALRDFMVVPQSCDKTTCVFKKLNIVSDKGVVDVKSFIKLLDKFTNSYPVWNSAKARVITTCLRKSLIAYDGGCELNNILACTFDVLSENCPLNGNNQTC

>BmorOBP41

GNIPEQPRVYCGELPNTIYSCLGNPKIIQPEVSEKCNKPISECDKTRCIFKESGWAKNNVIDKKKVSDYFEQFAKDNPDWSAAVQNFKTTCLSDSLKPQGVDTNCPAYDIIHCALISFIKFASPSQWSTSEQCVYPRQYAGACPVCPERCFAPSVPNGSCNACLALLRTP

>BmorOBP42

EDPPGLPPFLKDAPEKCKSPPRVKNPNECCISEPFFKEADFIECGIEKPGSERGPPDCSKQNCLLKKYNLLKNDETPDIEAIKSLLDKYIEKNPSFKSSVEKAKECLREDLPGPPQICLANRMTLCIGTVLLMECPDEKWNTTDDCKAFKDHMTECQKYFPK

>BmorOBP43

AKATLKPISACCNIPELGNPEPLAECSNPKLPGPCKDIQCVFEKSGFLTENKTLIKEAYKTHLRQWAKEHEGWSVAVEKAISDCVDKDLRQYLEFPCSAYDVFTCTGIAMLKKCPNEHWTC

>BmorOBP44

EFIINLYFNFITEIDSCCVKKYPKLFDSEFITECYNTQRKANDKCERDMCVARKLNLLTEEDSINKDALLRFVEEGFKTEIDLVNAIKKKCFEEDISNIGKPEMCEVAKYKICITSRMAEDCPKWDSKGICSSAQQKVENFMKMLS

>DmelOBP8a

VPMRSSPQSLALLRARDQCGRELTAAQRLQLDRMQFEDAAHVRHYLHCFWSRLQLWLDETGFQAQRIVQSFGGERRLNVEQALPAINGCNAKTSSRGSGAQTVVDWCFRAFVCVLATPVGEWYKRHMSDVINGNA

>DmelOBP18a

EGCLKHHNLTSAQVQAVAPSTPVADVPVAVKCYSRCLIQDYFGDDGKIDLQKVGKRGSQEDHVILSQCKQQFDGVTNLDTCDYPYLILQCYFKGKQSGTIAS

>DmelOBP19a

GVTEEQMWSAGKLMRDVCLPKYPKVSVEVADNIRNGDIPNSKDTNCYINCILEMMQAIKKGKFQLESTLKQMDIMLPDSYKDEYRKGINLCKDSTVGLKNAPNCDPAHALLSCLKNNIKVFVFP

>DmelOBP19b

DEEEGSMTVDEVVELIEPFGDACTPKPSRENIVEMVLNKEDAKHETKCFRHCMLEQFELMPEDQLQYNEDKTVDMINMMFPDREDDGRRIVKTCNEELKAEQDKCEAAHGIAMCMLREMRSSGFKIPEIKE

>DmelOBP19c

QTQAFDLAKLLPKTGTEPIWAVIDRNLPQVQELVTAARMECIQKLQLPRDQRPLGKVTNPSEKEKCLVECVLKKIKLMDADNKLNVGQVEKLTSLVTQDNKMAIAVSSSMAQACSRGISSKNPCEVAHLFNQCISRQLERNNVKLVW

>DmelOBP19d

KPHEEINRDHAAELANECKAETGATDEDVEQLMSHDLPERHEAKCLRACVMKKLQIMDESGKLNKEHAIELVKVMSKHDAEKEDAPAEVVAKCEAIETPEDHCDAAFAYEECIYEQMKEHGLELEEH

>DmelOBP22a

KEPEEVKIVSECAKENNVHRKKALDLLMSYRLKKKTHNVMCFINCIFERTNILQKVKEKVVKENHNCDSIKDADKCAESFQKFQCLVKIEMKRDSAATRHVPQTMPKL

>DmelPBPRP5

FDEKEALAKLMESAESCMPEVGATDADLQEMVKKQPASTYAGKCLRACVMKNIGILDANGKLDTEAGHEKAKQYTGNDPAKLKIALEIGDTCAAITVPDDHCEAAEAYGTCFRGEAKKHGLL

>DmelOBP44a

SDYKLRTAEDLQSARKECAASSKVTEALIAKYKTFDYPDDDITRNYIQCIFVKFDLFDEAKGFKVENLVAQLGQGKEDKAALKADIEKCADKNEQKSPANEWAFRGFKCFLGKNLPLVQAAVQKN

>DmelOBP46a

RSTPPALDEDCELNSVDTMHDFCCDLHDESPQFSDCQMEWHEKIPYETDEEEQTYMFCTAECSFNSTNFLGRDRRSLNLNEVKEHLESDLVNDADIKLLYDTYVKCDKHALSLMPHKGVKQLSKRLSRLGCHPYPGLVLECVANEMILHCPTKRFRQTAQCEETRNHLKQCMQYLKYKS

>DmelOBP47a

EININLGLTVADESPKTITEEMIRLCGDQTDISLRELNKLQREDFSDPSESVQCFTHCLYEQMGLMHDGVFVERDLFGLLSDVSNTDYWPERQCHAIRGNNKCETAYRIHQCQQQLKQQQQNLLATKEVEVTTTPAGSDETKP

>DmelOBP47b

QATIDCQRPPQLVDPALCCKDGGRDQVAEQCAQRILGTANGQKAGGPPSLDTAACLAECILTSSKYIDEPQKLNLANIRSDLSAKFSNDTLYVETMTMAFSKCEPQSQRRLAMIMQQQQQVQQQKTQQQQPRCSPFSAIVLGCTYMEYFKNCPDHRWTPNAQCTLAKAYVTQCGLGA

>DmelOBP49a

DVDCSKRPSFVNPKTCCPMPDFVTAELKQKCIKFDMTPPPPPDGEASGSFESKRRHHHPHPPPCFFSCIFNETGIYQNRKLDEAKLNAYLQEVFEDSSDLQTTATQAFTTCATKVADFEANLPPRPAPSPPPGFPMCPHDAGHLMGCVFRNMMKNCPDSIRNDSQQCTDMKEFFTKCKPPRGPPPSAEDM

>DmelOBP50a

AKCRAAPKSVQNVHVCCSAPLPNWGVFNRECHKSAIQASCRLDCDFNASSVLQGNRLIQAKVRPMLERAFSNEPTIDAYESNFAKCSTVVRSKYQELSPLSRQSDACDRHALFYSLCAYARLIFTCPDKMWQRNNRMCQEAKAYAKKCPWPALKMFMRNT

>DmelOBP50b

VSNDMGGLQKCTELLNTHKLVYCCGKSFLDKFPFVGSNCTPFWDDYGPCRYECLYRHWDLLDQDNKIKKPELYLMITSLYSPLNGYDKYGAAFKAAHETCEALGSRHADFLLLYSNQVADKMGMASSTCLPYAMLHAQCTMVYLTANCPRENWIDDPKCNSLQKLLSSCTKKLDEKTNALKGKDEELTDNGCGHIDSEGSNLLMACFLTLMIAKFISDH

>DmelOBP50c

DPIDVDCTRRQDFNIVKDCCVYPTFRFDQFKSQCGKYMPVGAPRISPCLYECIFNKTNTVVDGAIHPDNARLMLEKLFGNQDFEEAYFNGLMGCSDSVQEMISNRRSRPQRKTEQCSPFSLFYGICAQRYVFNHCPSSSWSGTESCEMARLQNMNCSKPSRGSSHRL

>DmelOBP50d

DPICSQRPDVTALRNCCKLPNLDFSSFNSKCSQYLVNGVHISPCSFECIFRAANALNGTHLVMENIEKMMKTILGSDEFVHVYLDGFRSCGNQEKVLIKAMKRRRVPITGKCGSMAIMYGLCAHRYVYRNCPESVWSKSATCNEAREYSIRCDDM

>DmelOBP50e

SFNCSAPPNFNNFDINTCCRTPELDMGDVPQKCHKYVSGLKSANSKYPSYAHLCYPDCIYRETGAMVNGKIKVNRVKQYLEEHVHRRDQEIVSHIVQSFESCLSNVKGHMKSLNIESYKVLPHGCSPFAGIIYSCVNAETFLNCPQQMWKNEKPCNLAKQFAEQCNPLPHVPLPSS

>DmelOBP51a

LFESEANECAKKLGITPDYFENFPHSSRVKCFYHCQMEKLEIIANGVVTPFDLKVLNISPESYDKYGVKVKPCLKLSHRDKCELGYLVFQCLKREFNL

>DmelOBP56a

SSLNLSDEQKDLAKQHREQCAEEVKLTEEEKAKVNAKDFNNPTENIKCFANCFFEKVGTLKDGELQESVVLEKLGALIGEEKTKAALEKCRTIKGENKCDTASKLYDCFESFKPAPEAKA

>DmelOBP56b

QSAAELAAYKQIQQACIKELNIAASDANLLTTDKEVANPSESVKCYHSCVYKKLGLLGDDGKPNTDKIVKLAQIRFSSLPVDKLKSLLTSCGTTKSAATCDFVYNYEKCVVKGISA

>DmelOBP56c

RSLSVSLNMSMTRTLVPDPPNGTENKLSQEMLRACMRRTEISMSQLKLFHMSLMNSDYNNDNDIAPTPVQSIGDCFVSCLYETLDLDRYNVLLEEAFKNQVQTIIQHEKAEIKECSDLQGKTRCEAAYKLHLCYNHLKTLEAEQRIREILERTEAENEGFGPEGSDFIDGIQHSGEAMTTAKSE

>DmelOBP56d

AELQLSDEQKAVAHANGALCAQQEGITKDQAIALRNGNFDDSDPKVKCFANCFLEKIGFLINGEVQPDVVLAKLGPLAGEDAVKAVQAKCDATKGADKCDTAYQLFECYYKNRAHI

>DmelOBP56e

QKAEAKQRAKACVKQEGITKEQAIALRSGNFADSDPKVKCFANCFLEQTGLVANGQIKPDVVLAKLGPIAGEANVKEVQAKCDSTKGADKCDTSYLLYKCYYENHAQF

>DmelOBP56f

MKSSEKIKACLKRQLGYTITENTKFDAKEDSLQSKCFYHCLLEVKGVIANDAISSEQPRKVLEKKYGITDTDELEKAEEKCHSIKASGKCELGYEILKCYQSITKH

>DmelOBP56g

QANIDSSVSKELVTDCLKENGVTPQDLADLQSGKVKAEDAKDNVKCSSQCILVKSGFMDSTGKLLTDKIKSYYANSNFKDVIEKDLDRCSAVKGANACDTAFKILSCFQAAN

>DmelOBP56h

NPDFRQIMQQCMETNQVTEADLKEFMASGMQSSAKENLKCYTKCLMEKQGHLTNGQFNAQAMLDTLKNVPQIKDKMDEISSGVNACKDIKGTNDCDTAFKVTMCLKEHKAIPGHH

>DmelOBP56i

GPIKDQCMAAAGITAQDVANRHETDDPGHSVKCFFRCFLENIGIIADNQIIPGAFDRVLGHIVTAEAVERMEATCNMIKSETSHDESCEFAWQISECYEGVRLSDVKKGQRTRNHRG

>DmelOBP57a

KESQPFDFFEGTYDDFIDCLRINNITIEEYEKFDDTDNLDNVLKENVELKHKCNIKCQLEREPTKWLNARGEVDLKSMKATSETAVSISKCMEKAPQETCAYVYKLVICAFKSGHSVIKFDSYEQIQEETAGLIAEQQADLFDYDTIDL

>DmelOBP57b

RHPFDIFHWNWQDFQECLQVNNITIGEYEKYARHETLDYLLNEKVDLRYKCNIKCQLERDSTKWLNAQGRMDLDLMNTTDKASKSITKCMEKAPEELCAYSFRLVMCAFKAGHPVIDSE

>DmelOBP57c

IQSLSLLEETNYVSDCLASNNISQAEFQELIDRNSSEEDDLENTDRRYKCFIHCLAEKGNLLDTNGYLDVDKIDQIEPVSDELREILYDCKKIYDEEEDHCEYAFKMVTCLTESFEQSDEVTEAGKNTNKLNE

>DmelOBP57d

NDPCPHNQGIDEDIAESILGDWPANVDLTSVKRSHKCYVTCILQYYNIVTASGEIFLDKYYDTGVIDELAVAPKINRCRYEFRMETDYCSRIFAIFNCLRQEILTKS

>DmelOBP57e

NTSVFNPCVSQNELSEYEAHQVMENWPVPPIDRAYKCFLTCVLLDLGLIDERGNVQIDKYMKSGVVDWQWVAIELVTCRIEFSDERDLCELSYGIFNCFKDVKLAAEKYVSISNAK

>DmelOBP58b

VRVHCRHMERIHEENIHHCCKHQDGHDDVTESCAKQTNFRLPSPNEEAIVDVTVDQAMVGTCWAKCVFDHYNLMENNTLDMDKVRSYYKRYHQTDPEYATEMLNAYEKCHTQSEEATEKFLSLPIVRAFSTAKFCKPTSSIIMSCVIYNFFHNCPASRWSNTTECVETLAFARKCKDVLTTM

>DmelOBP58c

IKIDCENTEAINEDHIHYCCKHPDGHNDLIEGCARETNFTLPNQNEEALVDITADRAIRGTCFGKCVFSKLNLMKDNNLDMDAVRSLFTERFPDDPEYAKEMINAFDHCHGKSEENTSMFLSKPLFKQMSKQFCDPKSSVVLACVIRQFFHNCPADRWSKTKECEDTLAFSKKCQDSLATL

>DmelOBP58d

QDNEETTAVAISSGDLTEDKCNTSRAGCCSELYIGEEEDLVKCFVIHSPKLPVDGDADIGKTLRFLSCFVECLYKQKKYIGKSDTINMKMVKLDAEKTFVDRPKEKDYHIAMFEFCRKDAVGVYNLLKASPGAKVLLKGACRPYLLMVFMCISDYHQKHECPYFRWEGTAKAGTKDMCENAKAECYQIDGITLPTKSPA

>DmelOBP59a

LKCRSQEGLSEAELKRTVRNCMHRQDEDEDRGRGGQGRQGNGYEYGYGMDHDQEEQDRNPGNRGGYGNRRQRGLRQSDGRNHTSNDGGQCVAQCFFEEMNMVDGNGMPDRRKVSYLLTKDLRDRELRNFFTDTVQQCFRYLESNGRGRHHKCSAARELVKCMSEYAKAQCEDWEEHGNMLFN

>DmelPBPRP1

VEINPTIIKQVRKLRMRCLNQTGASVDVIDKSVKNRILPTDPEIKCFLYCMFDMFGLIDSQNIMHLEALLEVLPEEIHKTINGLVSSCGTQKGKDGCDTAYETVKCYIAVNGKFIWEEIIVLLG

>DmelLUSH

MTMEQFLTSLDMIRSGCAPKFKLKTEDLDRLRVGDFNFPPSQDLMCYTKCVSLMAGTVNKKGEFNAPKALAQLPHLVPPEMMEMSRKSVEACRDTHKQFKESCERVYQTAKCFSENADGQFMWP

>DmelPBPRP3

QRDENYPPPGILKMAKPFHDACVEKTGVTEAAIKEFSDGEIHEDEKLKCYMNCFFHEIEVVDDNGDVHLEKLFATVPLSMRDKLMEMSKGCVHPEGDTLCHKAWWFHQCWKKADPKHYFLP

>DmelOBP83b

QEPRRDGEWPPPAILKLGKHFHDICAPKTGVTDEAIKEFSDGQIHEDEALKCYMNCLFHEFEVVDDNGDVHMEKVLNAIPGEKLRNIMMEASKGCIHPEGDTLCHKAWWFHQCWKKADPVHYFLV

>DmelOBP83cd

LLEHEGETINRCIQNYGGLTAENAERLERFKEWSDSYEEIPCFTRCYLSEMFDFYNNLTGFNKDGIVGVFGRPVYEACRKKLELPFESGESSCKHAYEGFHCITNMESHPFTVIDNMPNISPSAKDAMKDCLQDVHQDEWKSFDAFAYYPVNEPIPCFTRCFVDKLHIFEEKTRLWKLEAMKQNLGIPAKGARIRTCHRHRGRDRCATYYKQFTCYAMAV

>DmelOBP83ef

DLSGDAQTLEKCLRQLSSPESIAGDLRKLERYSSWTREEVPCLMRCLAREKGWFDVEENKWRLKQLTEDLGADVYNYCRFELRRMGSDGCSFAYRGLRCLKQAEMHAGTSLSTLLQCSRQLNATNVELLQYSKLKSKEPIPCLFQCFADAMGFYDPDGNWRLENWKQAFGPSGNEDQSSGADYSGCRLSGTQREVALSKCSWMYHEYKCWERVNGNKLVEDNEEQ

>DmelOBP83g

KFLLKDHADAEKAFEECREDYYVPDDIYEKYLNYEFPAHRRTSCFVKCFLEKLELFSEKKGFDERAMIAQFTSKSSKDLSTVQHGLEKCIDHNEAESDVCTWANRVFSCWLPINRHVVRKVFA

>DmelPBPRP4

LQDHAKDNGDIFIINYDSFDGDVDDISTTTSAPREADYVDFDEVNRNCNASFITSMTNVLQFNNTGDLPDDKDKVTSMCYFHCFFEKSGLMTDYKLNTDLVRKYVWPATGDSVEACEAEGKDETNACMRGYAIVKCVFTRALTDARNKPTV

>DmelOBP85a

NSECRKSLNLPAHRKFNFAELYTINMCIEECNFIGCGYIEIDPPFRLDLANIRTNLQTIAPQPQNESIPFLVDAYRKCELFRSSHGRRFTLHLPDIEFIEEPCNPFALQITICVRIHAMQKCPSEFYVDSDECRLAREYFTQCVGDIETNLA

>DmelOBP93a

CDVQKNDKAINSCRKSLLGNNSTNSNGEVRNLKSDKVALHACIAECSFRTNGFLLSNGTVNTQALQKSYQQRYKNDPNMSQLMLKSLNSCTDYARKRVQEFQWMPKKGDCDFYPATLLACVMEKVYINCPTSKWKNTSDCTAMWKYLVACDDVASNKKK

>DmelOBP99a

DYVVKNRHDMLAYRDECVKELAVPVDLVEKYQKWEYPNDAKTQCYIKCVFTKWGLFDVQSGFNVENIHQQLVGNHADHNEAFHASLAACVDKNEQGSNACEWAYRGATCLLKENLAQIQKSLAPKA

>DmelOBP99b

DHHHHHHDYVVKTHEDLTNYRTQCVEKVHASEELVEKYKKWQYPDDAVTHCYLECIFQKFGFYDTEHGFDVHKIHIQLAGPGVEVHESDEVHQKIAHCAETHSKEGDSCSKAYHAGMCFMNSNLQLVQHSVKV

>DmelOBP99c

DDWTPKTGEEIRKIRVDCLKENPLSNDQISQLKNLIFPNEPDVRQYLTCSAIKLGIFCDQQGYHADRLAKQFKMDLSEEEALQIAQSCVDDNAQKNPTDVWAFRGHQCMMASKIGDKVRAFVKAKAEEAKKKAA

>DmelOBP99d

ASVWKLPTAQMVYEDLEKCRQESQEEDAATLRCLVKKLGLWTDESGYNARRIAKIFAGHNQMEELMLVVEHCNRMEQDTSHLDDWAFLAYRCATSGQFGHWVKDFMSQKEVER

>DvirOBP1

DDMEETIEECVKSLGLPDNYVEKLKSQQLIDDKSPCFTKCVMEKRGLIDGEGKLNVDNAEKDIDSDEKASQKSKEIAKNCVRHITTISSCEDTLKYNACLSPVWCSDGTLAHRK

>DvirOBP2

EFMEKVTRFGEKCMEETKATSEDVSQIMAHKIPESHEGKCMISCVYKAFKIQNEDGTMNSDETLKLMERIKESDAELYENLIKVFKICHGKPELIVADPCLTAVNVGTCAVTEGKALGIKSELFGM

>DvirOBP3

VLDPKIVASFMEKVTKFGEKCMAETKATSDDVARIIAHQIPETHEGKCMVSCVYKAFKIQNEDGSMNPDETLKLMEQVKESDAELYEKLMKVFTTCQGKKDLIDEDGCVTAVNVAACAITEGRAAGIKSEMFGM

>DvirOBP4

GERREQLRTAFHECVSGESGGPNDPRKLVLQDDADVAKVGAAIFCINKKTGVQSENGDININVLKQDVSLWAKDDVKASEIVDECTKNKGADANETAFNVFKCLVKKNGK

>DvirOBP5

ANTERGRLSELVKECQKESGSTVNEIEIIRNNGVITPDTGKQALCVNRKFGVLDQNGDVIPDQFNQHIEILSGGNSDARKEFKDNCSKTEGENPEAKAIKFVKCMQTVLAKYPV

>DvirOBP6

MGNPERHRVQELVKECQKESGSTVNEIENIRKGVITTETGKQALCVNIKVGALDQNGDVVPDGFKKHVEALTDSIDGRKEFQDKCGKTEGKSSEDKAINFVKCMQSV

>DvirOBP7

LNEKQMEAAKKVVTNVCTQKTKATLEEIDKMHHGDWEIDHSAMCYMWCAFNMYKLMHKNNTLNYASATTQLNQLPESFYESAKMSIEKCKDEAKTLNDKCVAAYEIAKCMYFANPDKYYLP

>DvirOBP8

MPAHDMPEEEIEKMKKFHEECKAQTGVDMEEVKKSLKEGTPTDAIKEHTLCFIQKTGLVGDDNKIDIDLAKSHFESVFPELADTIMADCISDEEITKDSAYNMAKCVVGKIGH

>DvirOBP9

DRHDDIKAAAEACNLNKVVLQSQDKEKIGETVFCVNKKIGLMNDDGSLNENVFKSDAKLWNSDDDLTQKIFNNCKDLKGDTPTIKAYNLAKCIKENRN

>DvirOBP10

LNEKQMQAAIKVVTNVCSQKAKATLEEIEKMHHGDWEVDHSAMCYMWCAFNMYKLMHKNNTLNYASAVQQLTQLPDSYYPSAVLCTEKCKDEAKTLNDKCVAAFELSKCMYFCNPEKYFLP

>DvirOBP11

LDPEMQELADMLHNTCVGETGASEDDINNAKTGNFADKEEFKCYIKCLMAQMACIDDDGIIDEEATIAVLPEEFRDKAAPVVRKCGTKKGKNDCENAWLTHKCYQSEAPDDYFLV

>DvirOBP12

FTEEQQQLMDSLHAECVSQTGVGEDAIGKAKGGDFVDDPKLKCYMKCVFNEIGVITDDEKIDIEGALAILPDEMKDIATPVITKCGTQAGADVCEAIFNTLKCYYDMDKRAFFLP

>DvirOBP13

HPLDLSDFKQEVIELTKVLHNECVGKTGVNEQIIDKAKEGVFEDDVKFKKYLSCCWVTSGVMEKSGKLNEDMLIGFIPPKYKTTIGKSVLTCQKNVAGIKPLHNMVFEMEKCIYAAAPELFVMF

>DvirOBP14

RMELPPELQEYVKDLHNICIKKAGVSESDHAAYDIVNNPHDPKLMCYMKCLMLEAKWMSPDGAIQYDYIINMAHPKIKDLLEAALNKCRTIEEGGDLCEKASNLNFCLSKADPENWFLV

>DvirOBP15

EEMKAKIMDIQKECSAATGIDDQAVAQALSGNFPTDDKFKKFVLCFGKMIGFLDEEGNPNIAAISEAMKKNFGDQMSTDVIMGCTKKESTPEETAYNVFKCSFKVIHM

>DvirOBP16

APPARKTSAEKREDMKQRTVIGLSCLKETKLDRSVVEKAMAQLGHSEDPKYKDFLSCSYKKQGYQDENGNILYENISNFLSNYYKADSLKIVEKCKGTTGSNHSEMAYNAMRCVISTLNDIPDEELI

>DvirOBP17

AMDRSKFPPKILENLNEWHNKCKRMTGTTEADITEMINGRFPENIAIKRYLYCLWEIIIGIKKGFDIDVEQIWHYLPNMHKADYVNYALCHKKAKETPGDDWVEKIWQMQKCNQKRIDAEHYIFF

>DvirOBP18

AMSEKQINATKKLVRNTCMNKANVAPEIVDAMHKGDFSQGQCYISCIMNTYKLISPEGTFDWEGGIRTMEANAPKSLVATASVSIKKCKDAMKTSVKSNKCQGAAEIAQCIYEDNPPNYFFP

>DvirOBP19

LSICFFAVANCQLDKKDFGAHLLEVVDVTHDKCQRITGASQALIDEMKKGSFPEDIAMKRYTYCLWMLIMKLREDLVLDSRKLWYYVPDMHKEDAVVYMKCNEEARKLPGDDLVSKIWNMQKCIQKNIDDKHYIYF

>DvirOBP20

FHMPEHDRVQATIAECQKESGSSVDELENVKLGKINPGTGKQVLCVHRKLGFMDENGDILAGPYKQHVEIITFDKGRQQELQACSKPEGENAEAKAVNFDKCFQSVLQKMGRD

>DvirOBP21

APQSDIKELQNLLQALSENCARKAKISQHLATNIENGIFEDDAKSKAYVACNWLDSTVIDEQGNLNEDILDHLCPPSKKPQLTNLIIRCHKAQPKNQSVEDIIYGMSKCIYPEDTTLFKIK

>DvirOBP22

APQSDLKDLENLLQVLKENCVKKSKISKNLLANIKNGIFEDDAKSKAYVACNWLDSTVIDEQGNLNVDLVDQILPPSKKPQLSNLIIRCHKAQPKNQSAEDIIYGMAKCIYPEDSSLVI

>DvirOBP24

LKCEFGNNNGENVRQALSQCLNSNETEHFWKMAMMDESDEGSSEEGEHSMTNSMNGSNSSTVAKTKRAVDDKMTKPGTDTDSNTTEEPDNGDSNEACLIQCVFTNMDLMDTNGMPDHSKLLEGLLKTATSRELRNFFQDTVDQCYQELNEGNKMDSCSFSTKLVKCLVEKGKANCADWPAG

>DvirOBP25

NKLELNSSMTLVTCMEEAGMTFDQFKEQLKSNGHDALCVLKCSYEKTGALDKDGNVDPGVLWAHLVKHGLDDTPNIKNKFTECMKSAGKILTCDDAVKFASCFDDIFNF

>DvirGOBP1

REESQLTEEKMEEFFHFWREDFKFEHRELGCALQCMSRHFNLLTDSSRMHHENTDKFIKSFPNGAVLSKTMVELIHNCELQHDAEEDHCWRILRVAECFKISCMKAGIAPSMEVMMAEFIMETENK

>DvirOBP27

MDSSTEASFIGDKIRQTTNILLGNCRSKTGATKEDFETLRERKIPTTKTGLCLMDCLFEGAGIMENGKFVRSGMVTSLTKAMKGDEAKIAKLNELGKVCEKELDNKQLPECQIGKKILECLARNGEKYGLEFVNPKT

>DvirOBP21a

KKAKISQHLATNIENGIFEDDXXSKIDEQGNLNEDILDHLCPPSKKPQLTNLIXXCHKAQPKNQSVEDIIYGMSKCIYPEDTTLFKIK

>DvirOBP28

EEDSSEEVTLTKQQVFDYLKILKKCELDSGAKPKYIRNSIMGRFKPEPKFKDYLYCLGNSTGLLDKEGNIDKKAFLHKTNSLIKDKEQAEKIVNSCLSQETAKPTAIFNTLTCYNGMRSKI

>DvirOBP29

ESNKVNLDPSISLVPCLEEAGMTFEQLIEKLKGMSHDALCVVKCSFVKAGTLDKDGNVDVNLVWTTMEKHGLLRPEIKTKFTECLESAGKILSCDDAEQHAYCFRDVFNF

>TcasOBP1

EDDDRQETIRQYRDDCIAETKVDPALIDRADNGDFTDDAKLQCFSKCFYQKAGFVSETGDLLFDVIKDKIPKEANREKALAIIDKCKELKGADSCETVYLVHKCYFLHSYGTDKKTE

>TcasOBP2

GLDPKFLEKLTQEVQAVGTSCGEKEHATADDMIEIMEEKFPPTSHEAKCVVACFYKHYKMMKEDGTFDKDAAVKAFDEIKAQDAEIHAKILKVIDACDAKKQMSDDHCNGLTKEAFMAS

>TcasOBP2a

RSFSHDELDTDLSFIKTCNRTSPISMSKFGLFLTEFNLTEPGTMNEVLINKKLGHGESSAFKCFLHCLFMKYGWMDSDGGFLLHDIKQTLEESDVEIASLEFILYKCTATESNNRCERAFVFTQCFWDKMAEQQPSEDQFFYNIEDKK

>TcasOBP2b

EKESEEAQIFTELDGPAAELRDQCLEKNSMKVTDLKTYNTSNDIPEKELCFYKCFYEGVEFIDANGNLNVNNMKEIPAISELGDEVLNEITACVEKIGKIRCCGDLRKIEQCYQNITM

>TcasOBP3

QKKGKYWTTISECLTEHSMGVEDMKKFDLPAEKMSEEMLCFNKCFYDKLLITDENGEINTDNLMSIPLVNAIDASKHDDLVTCLKKVGKIEECDGVKKIEQCFVEFI

>TcasOBP4

LDVEKIRNELMADKNFVELRNKCLDKLGLKEEDLRDLKFDGDVSEDLMCFGKCIQEEDGLLDSEGNLNEEKLEKKIETMPFLSRVSDDTKNNIMECLKEIGKIETCQDFGKQRDCIHKYV

>TcasOBP5

YFFMSQKFAEVREECLSENSMTMDELHEGWKMENLPESHLCFLKCLLEKREVIDENGVPQKEKIDEILTVKQLSDEKREEISTCITNVEKIENCETMSEIMRCFPKKRRD

>TcasOBP6

ISEEMQELANTLHATCVDETGVSEDAIESARKGNFAPDDKLKCYMKCIMEQMACIDDEGIIDVEATIAVLPEEYQAKAEPIVRKCGTKIGANACDNAFLTNKCWYEEDPEDYFLV

>TcasOBP7

IEMDDDMKELINNLHNTCTGETGATDDQIENARKGNFAEDDSFKCYFKCVFDQMGCMTDDGKVDSEAVIAVMPPELADKIASTVRGCTEVGANPCETAWLANKCYQKSNPDMYFVP

>TcasOBP8

EEMQELVNQLHSTCVAETGVSEDLINKVNSDKVMIDDEKLKCYIKCLLTETGCISDDGVVDVEATIALLPEDMKAKTTPVIRSCGAKMGANPCESAWLTHKCYLETSPADYVLI

>TcasOBP9

AMSEAQMKAALKLVRNVCQPKTKATNEQIEAMHTGNWDLDKNGKCYMWCILNMYKLIGKDNSFDWEAGIATLKAQAPESVRDPAIASVNNCKDAVKTTSDKCEAAYEIAHCMYLDNPEKYFLP

>TcasASP

ELDKEFLMQFLQKIKKVSEDCIAETQATKNDIKTLLEHKIPDSHEGKCMIFCFHKHFQIQNEDGSLNKVAAISLLEPIKDHSQDIYDKVVKIFNTCFDSAERDDDSCIYASNLAECAIRESKSLGLDDLLVIE

>TcasASP1

LSQDFIDKFVAKVKSIGETCVPETNASKDDISSLLAHKMPDSHEGKCLIFCFHKQFQIQNDDGSINREGAIKALEPLKADDAELYEKLLLTGIPVFMPLVWPNALSKKEELWDSTI

>TcasASP2

MEKIGEECAEETHATSDDIADLIEQRDPKTHEGKCLIFCYHKKFNTMKEDGSLDKVGSVLALEEVRDADFELYKNILTIFVTCGDKAKIYDDPCETATALTMCGRDEAKADAIFG

>TcasASP3

QEFVDEFLEKMQEFGAQCAEETDATSDDIAELIARKLPPSTHEGKCMIFCMQKKFNMMKENGGIDRAGAIAALKPLQKADPELHQKVLKIFVTCGMRVKPSPDPCDTATELALCGKKEAEAIGLEDALLT

>TcasASP4

MDESFLQQTRDRVKAIVKECVTEEKATDSDFDDIMALKIPTSHEGKCVFFCSHKKFNMQHPDGSINKEGALDTFEVVKDVDAEFHDKVITVYNHCLSTPVDPDPCVYSVNLFQCFMKEAKAAGIHELIIK

>TcasPBP

EDLINKVNSDKVMIDDEKLKCYIKCLLTETGCISDDGVVDVEATIALLPEDMKAKTTPVIRSCGAKMGANPCESAWLTHKCYLETSPADYVLI

>TcasOBP12

IDKEFVQELRQKLRSHVEACAKEVNAGPDDVSAIFAHKLPATHEGKCIFFCMHKLYNAQNEDGSLNMAGALANLELIKDMDPDVYTKVSTSFKNCESAPFDSDPCLYAANLVTCIVKEGRAVGLDEVLVE

>TcasOBP13

MDESFLQQTRDRVKAIVKECVTEEKATDSDFDDIMALKIPTSHEGKCVFFCSHKKFNMQHPDGSINKEGALDTFEVVKDVDAEFHDKVITVYNHCLSTPVDPDPCVYSVNLFQCFMKEAKAAGIHELIIK

>TcasOBP14

ELDKEFLMQFLQKIKKVSEDCIAETQATKNDIKTLLEHKIPDSHEGKCMIFCFHKHFQIQNEDGSLNKVAAISLLEPIKDHSQDIYDKVVKIFNTCFDSAERDDDSCIYASNLAECAIRESKSLGLDDLLVIE

>TcasOBP15

QSLSEDEMRENARKLMTSCKDKVGASDADVEALKMHQMPESREGFCMLECVFDSAKIMQDGKFSKSGMIEGFKPLIGDDKAKLESLEKLSATCESELGDGEDKCETAKRLVECVIKNGKTHGFEVPPPRE

>TcasOBP16

GLDPKFLEKLTQEVQAVGTSCGEKEHATADDMIEIMEEKFPPTSHEAKCVVACFYKHYKMMKEDGTFDKDAAVKAFDEIKAQDAEIHAKILKVIDACDAKKQMSDDHCVSAASMAGCVKTEAIAV

>TcasOBP17

QEFVDEFLEKMQEFGAQCAEETDATSDDIAELIARKLPPSTHEGKCMIFCMQKKFNMMKENGGIDRAGAIAALKPLQKADPELHQKVLKIFVTCGMRVKPSPDPCDTATELALCGKKEAEAIGLEDALLT

>TcasOBP18

VDQEFVEKFLQKMEKIGEECAEETHATSDDIADLIEQRDPKTHEGKCLIFCYHKKFNTMKEDGSLDKVGSVLALEEVRDADFELYKNILTIFVTCGDKAKIYDDPCETATALTMCGRDEAKALGLQDAIFG

>TcasOBP19

LSQDFIDKFVAKVKSIGETCVPETNASKDDISSLLAHKMPDSHEGKCLIFCFHKQFQIQNDDGSINREGAIKALEPLKADDAELYEKVISIFKKCESTPVDGDSCLYAASLAECAVKEGRAMGLDNLIVLEIE

>TcasOBP20

ENEHEILEVRALCMNETGVSEETARNYKPAEDPASEEILCMVKCIFEKIGCLKDDGSFCVDTMKKKNYIMDVINEENEEKIYECLRGVGKITNCRDMAAVEECFVKNDS

>TcasOBP21

CMKKLSVGETELAKALEDKSKDPPEKIMCLFKCALEDSGFLQDGVVDKSKWPMPECVQDVVKITNCNDMVALKHCFD

>TcasOBP23

AMSEAQLKAAVKLVRNMCQPKSKATNEDIEKMHHGDWNIDRTAMCYMHCALNSNKLITKENVFNRDYAITLAEKNLPTALKTASIEAANLCKDSAKTLDDKCVAAYEISKCLYESNPEKYFLP

>TcasOBP24

EIVVPDDLKDYINELHDHCLKEMGLTEGDHKNYNIHVKDPKMMCYMKCLMTTSKWMNMDESIQYDFILSSVHPAVKNILLPALDKCRDIPKGTMECEKAYNFNMCLFNADPENWFFI

>TcasOBP25

EIDGYYDICYKQIGLTKDDLKAYKIGDRDPKIMCFMKCVFVEAKWMDENENLQYDYIKNTIHHSIRHITLPELENCGKKAEGDKCEKSFSFFNCMNKAEPEDWVLIQ

>TcasOBP26

EIDEYFEQCFEPNGVTMDDIKAYKMGDKDPKIMCFMRCLFVSGKWMDENENMQYDYIKETIHHAIRHITIPELENCGKEAQTGDKCEKSFNFFMCMNRAEPEV

>OasiOBP1

MWARLSDCAALLLLLASAARAWDVNMKLTGRIMDAAKEVDHTCRTSTGVPREMLHRYADGQTVDDDDFKCYLKCIMIEFNSLSDDGVFVLEEELENVPPEIKEEGHRVVHSCKHINHDEACETAYQIHQCYKQSDPELYSLVVRAFDATIGDD

>OasiOBP2

MKAAMAPLATATAAMLLLLAAAVRGQDDEMREMMDQLHQTCVGESGVSEGNIDAARKGNFIEDANLKCYMKCIFVQMTCMSDDGVFDADTAIAMLPDNLKDVASKALNACKGEKGSDACDTAFKINQCLFKQAPKDYILV

>OasiOBP3

MDKASAAAATAFLLIAVAALHAQALSLEQLRQTSKIVRNMCLKKTGVDLALVEGIQEGQFPDNQDLKCYMKCCMGAMQVLRQGRYNVNAAKNQAEKMLPPDLKDRFLSMLDACSDRGDGADDDCEMAYQLTKCSYETDKEIFLFP

>OasiOBP4

MRTSAAAAAAATGAALLVLAAVASAMEMTPEFMEIVNKCKTEHEPTEDELKGMMALKVPESSNGKCFMGCVLQEIGVVKDGKFDKEEAKKHAAAKMTDKDELEKHMQLIEKCSQEVGGETDSCGIGPKLMECIKQFAPEFDIALPKPSE

>OasiOBP5

MRTYLTLVFAAAALFAVAKADAEKVKEAVEKCKSSENLDSLDGLKSNKAPSTEEEKCFIGCMMMDMKLLSSDGQYDAASTKEMINSCEYLKDKPDEKSAALEVADDCAGKATGCSGHCECGPKAVGCLINGMVDKGYEESFARIDKMLQNLE

>OasiOBP6

MGAAVAAAVLLLVAVTNAEDSLMEIVIREVKGCMESEHLNSIGDLRSYNDASSPEQKCFLGCMLKKFKALDADGQYDAEGLKATIEHCPRMKALPNVQKAALQVADECAGKVTGCSDYCSCAPLAAKCLHEGMKNKSFQTIFIALDEALDKMQS

>OasiOBP7

MRTILPLAVSAMLLVAPSKTHEQDFTKGISDVKVCMASENLGSLDGLRANKEARTAEEKCFIGCLMKFVEVLNSDGQYDVALFKDHINRSPDLAKMQQKKAALLEVADSCAGKASACSGHCECGVIVANCLAEGMEAKGEETIYDLLEKIFAKMDA

>OasiOBP8

MQAPQLLLAALALCLSAAVAAQQAPWCPTTASQGVQEDMGQCAEEIKDAILREYAKTVASRRTRSAEMSEEDRLLVGCMVSCLFRKGPHSRLQTGSKLALAELGAMRLFSDGADDARYRNATATAVRRCSASSRSLLPDDGGPRHECELGFFMFECVSDQITEYCQWQPE

>OasiOBP9

MSALFTCCVAAWLLMAAALLQPTKGDEVWHNTDIPATMAECNATFRLGWRCWDNLLSDGHVIDESKYQQKCWFYCLLDKTGAMHADGAFDKDLLKTVLQGFPNGSSLAHLDETTYTCVAQRNEVDLCERAYAVVKCIMTEELSRMHHSS

>OasiOBP10

MKALLVACVAALGCLAVAVAAISESMSRAEEAAAKIDLPELFEECNETFTTPKATLNYFFSHGRLQNENDYGSKCFIHCLTDRSGEIDSDGNFDVDLIKVMTRRFPNETNIEGLNEMVETCVADRGETDFCERAYGLVSCLIKEKLTRLGHSH

>OasiOBP11

MSLAARLFSVTLLLAPVLFSDISTAGEVFTMSQIKAAVNECNDTYFLSQKNWDSVFTTGSLEDEKDLVAKCFFECVLEKTGAMDEKGTINSDITKAVFLASHEGTGTPVQGHDELIDMCVPGRDETDICEKGYALVKCVTLEELSRRHARK

>OasiOBP12

MFYFYAFTLCCLLWVLFHCSVNCVDIDIETIWRECNETFPASEESLISFGKNGTIPDENDSTARCFADCYGKKTTMLTSDGSLNWTTLDFIMRSYNMKPTATETFGKCQKDTSNVECMKSYLSLRCVAETIASLSNIR

>OasiOBP13

MVNHHQGVVAIAAALTAMAAAAPSSIAEATRFSKETVSKCQEKWQVSEEIIEEMQRNKGALPNEDSVEQRCFAECVAKEMGMINNGGGVAADKIVKMLEAVFQMASKETGEKLKLDSRALKRDLEACQFKGEDDECTNSYDTLKCLRTLGTSDNMRRYVTKES

>OasiOBP14

MFYFYAFTLCCLLWVLFHCSVNCVDIDIETIWRECNETFPASEESLISFGKNGTIPDENDSTARCFADCYGKKTTMLTSDGSLNWTTLDFIMRSYNMKPTATETFGKCQKDIPTSHQLKTLCTSLSLKHVNPSHQFHRGRGHGWLLKHVNRGPCTQRCKYT

>OasiOBP15

MRTSHVYTIFCAIIVTCYCDSVEVSDGPEEATMMKCAVELGFGHDEIQRIKSSPIPDETNENERCLMKCIGRKMKYLTSEDIVDVHHLLELSGEMIEKEGYTKSEMRQMLVECTKKTGTEKCMTAFKNLRCLMNAFK
