# Supplementary material for: Expressions of Olfactory Proteins in Locust Olfactory Organs and a Palp Odorant Receptor Involved in Plant Aldehydes Detection
Source: Front Physiol. 2018 Jun 4;9:663. doi: 10.3389/fphys.2018.00663 (PMC5994405; doi:10.3389/fphys.2018.00663)
Supplement: TABLE S6 — Sequence of primers used in RNAi experiments. T7 promoter is shown in red. [file Table_6.DOCX]

**Table S6.** **Sequence of primers used in RNAi experiments. T7 promoter is shown in red**

| **Primer name** | **Primer sequence** | **Purpose** |
| --- | --- | --- |
| T7-Lmig OR12-For | GGATCCTAATACGACTCACTATAGGCAGAGCGACTTCAAACC | RNAi template |
| T7-LmigOR12-Rev | GGATCCTAATACGACTCACTATAGGGCAAGGCTACACTCAAATAC | RNAi template |
| T7-GFP-For | GGATCCTAATACGACTCACTATAGGCACAAGTTCAGCGTGTCCG | RNAi template |
| T7-GFP-Rev | GGATCCTAATACGACTCACTATAGGGTTCACCTTGATGCCGTTC | RNAi template |
